# pK Values of the Cofactor Tune the Redox Regime of Flavoenzymes

**DOI:** 10.3390/life16081277

**Published:** 2026-07-31

**Authors:** Wolfgang Nitschke, Simon Duval, Kilian Zuchan, Jostin Monge-Ruiz, Frauke Baymann, Barbara Schoepp-Cothenet

**Affiliations:** 1 BIP (UMR 7281), CNRS, Aix-Marseille-University, 13009 Marseille, Francejmonge@imm.cnrs.fr (J.M.-R.); schoepp@imm.cnrs.fr (B.S.-C.); 2 Department of Inorganic and Analytical Chemistry, University of Geneva, 1211 Geneva, Switzerland

**Keywords:** flavoenzymes, redox cooperativity, hydrogen bond, flavodoxin, ETF, FNR, Ndh-2, SQR, electron bi/confurcation, pK values

## Abstract

Across the diverse family of flavoenzymes, the isoalloxazine cofactor was found to display extremely diverse redox properties, both with respect to the absolute value of the potential regime wherein it operates and to its redox cooperativity, that is, the relative positioning of its individual 1-electron transitions. Taking together electrochemical data and 3D structural information reported for selected representatives of the flavoenzyme family, we assessed the contribution of pK value modifications at the three protonatable nitrogens of the isoalloxazine moiety. While the absolute value of the redox regime appears only weakly dependent on such pK modifications, the diversity of redox cooperativity is readily rationalized by (protein-induced) stabilization/destabilization of the proton primarily on N5 and to lesser degrees on N1 and N3. The mathematical formalism underlying the interdependence of pK values and redox midpoint potentials is subsequently extended to representatives of the family featuring extremely positive redox cooperativity (i.e., the electron bi/confurcating flavoenzymes). Observed structural idiosyncrasies of these cases were found to rationalize the extremely strong inversion (ΔE ≪ −800 mV) of 1-electron midpoint potentials in the framework of this formalism.

## 1. Introduction

Flavoenzymes represent an extraordinarily vast and diverse group of redox proteins involved in a plethora of catalytic reaction schemes and electron transfer processes. The first flavoprotein, then called lactochrome, was purified from cow milk almost 150 years ago [1], and the structure of its pigment, called riboflavin (also known as vitamin B2), was deduced in the early 1930s [2,3,4]. The cofactors of all flavoproteins, i.e., flavin mononucleotide (FMN) and flavin-adenine dinucleotide (FAD), are derivatives of riboflavin [5]. Over the last century, flavoproteins were found to be elements of virtually all metabolic pathways both in microorganisms and in complex life [5,6,7,8,9,10,11,12]. In these pathways, they were observed to perform both 1-electron and 2-electron redox reactions or even to serve as gates mediating between 1-electron and 2-electron redox compounds. In addition to this functional versatility, the redox range covered by flavoenzymes (as defined by the average potential of their two redox transitions) extends from about 0 mV (vs. the standard hydrogen electrode) in electron-transferring flavoproteins (ETFs) [13] to −400 mV in certain flavodoxins [14], that is, 200 mV above and below, respectively, the value determined for flavins in aqueous solutions [15].

A novel functional subclass of flavoenzymes standing out by their exceptional redox reactivities was discovered in Wolfgang Buckel’s and Rolf Thauer’s groups in 2008 [16,17]. The particularity of this subclass consists in their ability to perform the process of electron bifurcation [18,19,20,21,22], a mechanism previously considered to occur only in mitochondrial complex III (and its homologs) [19,23,24,25] and to depend on the presence of quinones. In electron bifurcation, the two reducing equivalents harboured by 2-electron redox compounds (such as quinones or flavins) are transferred to two distinct 1-electron acceptors with dissimilar reduction potentials. In most biological electron bifurcating systems investigated so far, one of these 1-electron acceptors was reported to feature a lower redox midpoint potential than the 2-electron donor [17,26,27]. The reverse reaction is called electron confurcation and allows a 2-electron acceptor to oxidize two 1-electron donors, one of which may be at a higher potential than the acceptor.

Electron bifurcation and confurcation therefore expand the range accessible to biological redox reactions beyond the extent offered by the environmental redox substrates [18,28] and can be considered a biological counterpart of electronic DC-to-DC converters [29]. Electron bifurcation and confurcation have been shown to be evolutionarily ancient processes and may well have been part of the free energy converting mechanisms that thermodynamically drove the emergence of life on our planet [19,22,30,31,32,33].

The functional mechanism underlying flavin-based electron bifurcation is still not fully settled. Hypotheses relying on the electrochemical properties of the 2-electron redox compound [19,22], on conformational gating [34], on kinetic constraints [35] or on a combination of all these [36,37] have been proposed. For the case of the quinone-based electron bifurcation process, a very strongly positive so-called “redox cooperativity” between the individual electrons (corresponding to quasi-exclusive 2-electron redox transitions) has been recognized as crucial to the mechanism, and a detailed rationalization of this process has been put forward [24]. Negative redox cooperativity in 2-electron compounds refers to situations where the second reduction step occurs at a more negative ambient potential than the first one, resulting in a regime of ambient potentials where the 1-electron- (also called semi-) reduced state is stabilized. Positive cooperativity, by contrast, inverts the individual 1-electron potentials and results in destabilization of the semireduced state and in seemingly concomitant 2-electron transitions of the compound [19,38]. The term “redox cooperativity” has therefore historically been coined to account for the apparent hindering or favouring of the second reduction step by the presence of the first electron on the redox centre.

The strength of redox cooperativity forms a continuum, and a multitude of strongly differing cooperativity parameters have indeed been measured both in flavoenzymes and in quinone-based redox enzymes [22]. Just as for the case of quinone-based electron bi/confurcation, the flavin cofactor of electron bi/confurcating flavoenzymes has also been found to feature strong positive redox cooperativity [13,27,39,40]. We therefore consider (as we have suggested in the past [19,22]) that strong positive 2-electron redox cooperativity is a necessary condition for electron bi/confurcation to occur.

While quinones in aqueous solutions and at physiological pH values always feature very strong positive redox cooperativity and hence strict 2-electron redox transitions [41], the cooperativity of flavins under the same conditions is much weaker, and semireduced intermediates are readily observed [42,43,44]. This raises the question of how the protein environment in electron bifurcating flavoenzymes coaxes the cofactor into undergoing strongly cooperative 2-electron transitions. Case-by-case analyses of 3D structures of individual electron bifurcation flavoenzymes have addressed this question [27,39,45] but failed to discern common structural features [46].

As mentioned above, protein-harboured flavin cofactors occur with extremely variable degrees of redox cooperativity (both positive and negative) in different members of the entire family of flavoenzymes [22]. The redox properties of the flavin in many of these representatives have been characterized in great detail [17,27,47,48,49,50,51,52,53,54] (although the pH dependence of the respective redox midpoint potentials unfortunately was determined only in a few cases, see below).

In this work, we set out to use this large inventory of examples to elucidate a single overarching principle for tuning the redox properties of the flavin cofactors in these enzymes. Our approach represents an extension of the working hypothesis suggested by Schopfer et al. [55] using a larger sample of examples and applying a mathematically more rigorous treatment. In short, while Schopfer et al. approximated the experimental E-vs-pH curves of flavins in aqueous solutions and of the flavin cofactor in flavodoxins by straight lines featuring 0, −30 and −60 mV/pH dependencies, mostly using a single (the most obvious) pK, our analytical treatment takes into account the full set of nine pK values and allows for a generalization of Schopfer et al.’s working hypothesis towards the entire family of flavoenzymes. The analytical approach furthermore allows us to visualize how variations in pK straightforwardly move the cofactor’s redox behaviour back and forth between negative and positive redox cooperativity. Last but not least, evaluating the full formula for pH-dependent redox midpoint potentials permits the deduction of the potential values for the individual transitions even for cases of highly positive redox cooperativity. Our results demonstrate that redox cooperativity is much more about dissimilar electron-proton couplings of the two redox transitions than about interactions between the two individual electrons.

## 2. Materials and Methods

The theoretical pK- and pH-dependent E1 and E2 potentials were calculated via a C++ routine that wrote the respective pH, pKs, E1 and E2 values to a file, which ultimately served to generate 3D plots using Gnuplot (version 5.4., copyright 2007 Thomas Williams, Colin Kelley). Briefly, the C++ routine iterated pK(N1), pK(N3), and pK(N5) for all three redox states in nested loops, followed by sorting of the respective pK values into pK1 to pK3, again for all three redox states. The sorted pK1–3 values for the oxidized/semireduced states and the semireduced/reduced states were used to calculate E1 and E2, respectively, using Formula (3). C++ routines and Gnuplot were executed under Linux. Source codes of the C++ routines are provided in the Supplementary Materials.

## 3. Results and Discussion

### 3.1. A Brief Summary of Relevant Electrochemical Principles in Proton-Coupled Redox Transitions

**Figure 1:**
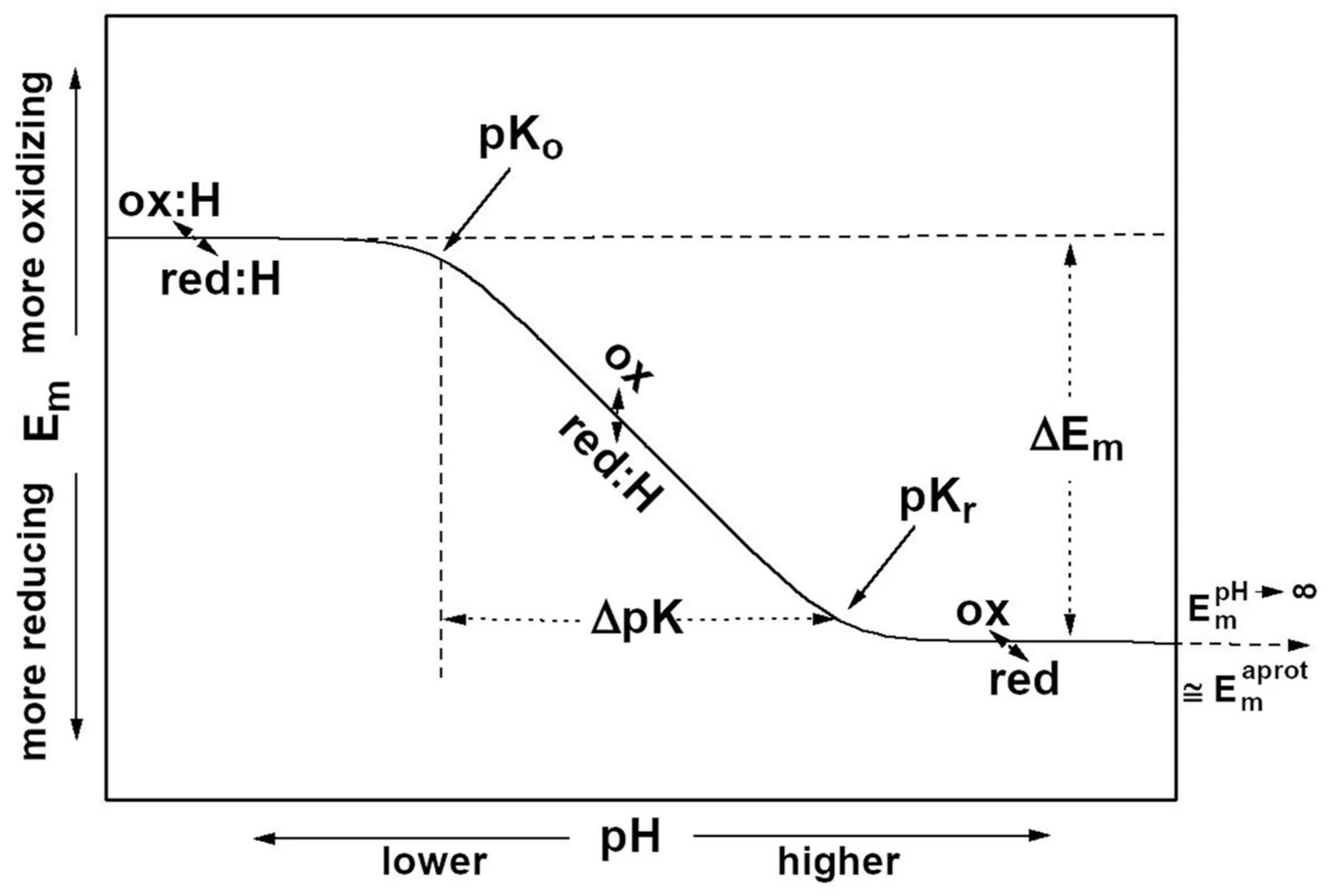
Schematic representation of the theoretical dependence of a protonatable 1-electron compound’s redox midpoint potential on pH value according to Formula (1). The significance of the relevant parameters is indicated in this scheme.

If a redox compound is in proximity to a protonatable group, its redox potential will depend on the protonation state. The protonated compound will obviously be easier to reduce (due to the presence of the positive partial charge afforded by the proton) and its E_m_ therefore will be higher than when it is in the deprotonated state. Conversely, the compound will be easier to protonate when it is in its reduced state, and the pK of the reduced state, pK_r_, therefore commonly is higher than that of the oxidized state, pK_o_. Figure 1 schematically depicts the E_m_/pH dependence of a hypothetical protonatable redox centre and indicates the relevant redox and protonation states, as well as the pK values characterizing the transitions between the protonation states of the oxidized and reduced redox centres. The E_m_/pH dependence of a 1-electron redox centre [56] is described by Formula (1)

(1) $$E_{m}=E_{m}^{pH\to \infty }-\frac{RT}{F}*ln[\left(1+\frac{a_{H}}{K_{o}}\right)/\left(1+\frac{a_{H}}{K_{r}}\right)]$$

wherein a_H_ corresponds to the activity of the protons (a_H_ = 10^−pH^) and K to the acid-dissociation constant of protonatable sites (R, T and F stand for the universal gas constant, ambient temperature and the Faraday constant, respectively).

The layout of Formula (1) differs from its version found in most textbooks (e.g., [57]) by using $E_{m}^{pH\to \infty }$ (e.g., [58]) as the scaling constant rather than $E_{m}^{pH=0}$. Whereas pH = 0 corresponds to standard conditions in chemical nomenclature, it is an arbitrary parameter (rather inspired by mathematical considerations than by a deeper chemical meaning). By contrast, $E_{m}^{pH\to \infty }$ represents a meaningful chemical property, i.e., the reduction potential in the absence of solvated protons, a value that may be empirically approximated in aprotic media as $E_{m}^{aprot}$.

The magnitude of the difference in redox midpoint potential between the protonated and the deprotonated compound, E^deprot^ − E^prot^ (and correspondingly pK_o_ − pK_r_ for the difference in pK between the oxidized and the reduced state) depends on the strength of the interaction energy (electrostatic, conformational etc) between the proton and the redox centre. For strongly proton-coupled redox reactions, the theoretical relationship [59] between ΔE and ΔpK is provided by the simplified Formula (2).

(2) $$1.37*[{pK}_{o}-{pK}_{r}]=23.06*[E^{deprot}-E^{prot}]$$

Empirical data are nicely in line with this theoretical prediction. For example, for the case of the [2Fe-2S] centre of the Rieske protein with protonatable sites on the nitrogen atoms of the two histidine ligands coordinating one of the irons of the redox centre, the empirical values for the proton with the lower pK have been determined to be pK_o_ − pK_r_ ≈ 5 and E^deprot^ − E^prot^ ≈ 280 mV [58,60]. Similarly, the proton residing on the hydroxyl group of benzoquinone-type moieties in aqueous solutions features values in the range of pK_o_ − pK_r_ ≈ 5–6 and E^deprot^ − E^prot^ ≈ 300–350 mV [41]). The interdependence of redox and protonation reactions is a general electrochemical principle, not restricted to biological entities, and has, for example, also been observed in inorganic (mineral-based) redox systems [61].

### 3.2. Free Flavin in Aqueous Solution

While the above-described formalism applies to single-electron transitions, flavins feature three reduction states, hereafter denoted as the oxidized (o), semireduced (sr) and fully reduced (r) states, and therefore belong to the class of 2-electron compounds (also encompassing, for example, quinones and molybdopterins). Depending on the mutual positioning in redox potential of the o/sr- and sr/r-transitions, flavins can undergo two consecutive 1-electron redox transitions involving the sr-state, 2-electron transitions directly between the o- and the r-state, or intermediate situations where samples become heterogeneous, with populations performing either consecutive 1-electron or concerted 2-electron transitions (see [22]). For 2-electron compounds such as flavins, quinones and molybdopterins, the formalism described in Section 3.1 applies to each redox transition (i.e., o/sr and sr/r) independently.

**Figure 2:**
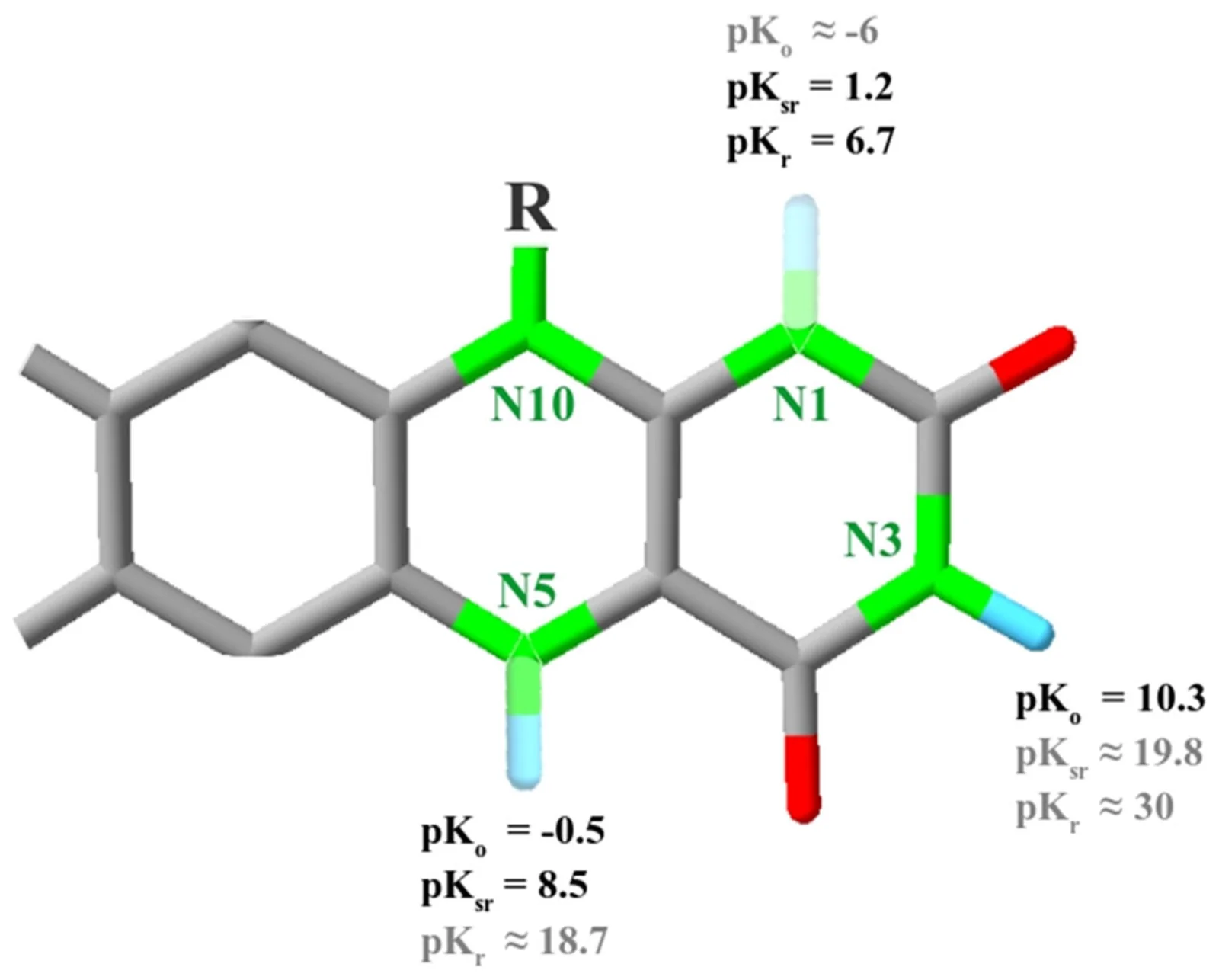
Chemical structure of the isoalloxazine moiety, i.e., the electrochemically relevant part of flavin-molecules (riboflavin, FMN, FAD, etc.). The pK values for the three protonatable nitrogens in each of the three redox states indicated in black print represent those experimentally obtained for flavin in aqueous solution. The values in grey print are deduced from measurements in aprotic media, assuming $E_{m}^{aprot}$ = $E_{m}^{pH\to \infty }$ using Formula (3) as detailed in the text. Green stands for nitrogen atoms, red for oxygens and blue for protons in this and the following figures featuring 3D structures.

As depicted in Figure 2, the isoalloxazine moiety, i.e., the core structure of all flavin pigments from riboflavin to flavin mononucleotide (FMN) and flavin-adenine dinucleotide (FAD) features three protonatable sites at the nitrogen atoms N1, N3 and N5. In the oxidized state and at neutral pH, only N3 is protonated, while N5 takes up a proton upon reduction of the flavin to the semireduced state and N1 becomes partially protonated upon 2-electron reduction (the attribution of the three successive protonations to positions on the isoalloxazine moiety is based on NMR measurements [62,63,64]). This basic observation already indicates substantially different and redox-dependent pK values for the three protonatable sites. This empirical rationale for the specific pK values indicated in Figure 2 will be elaborated below.

**Figure 3:**
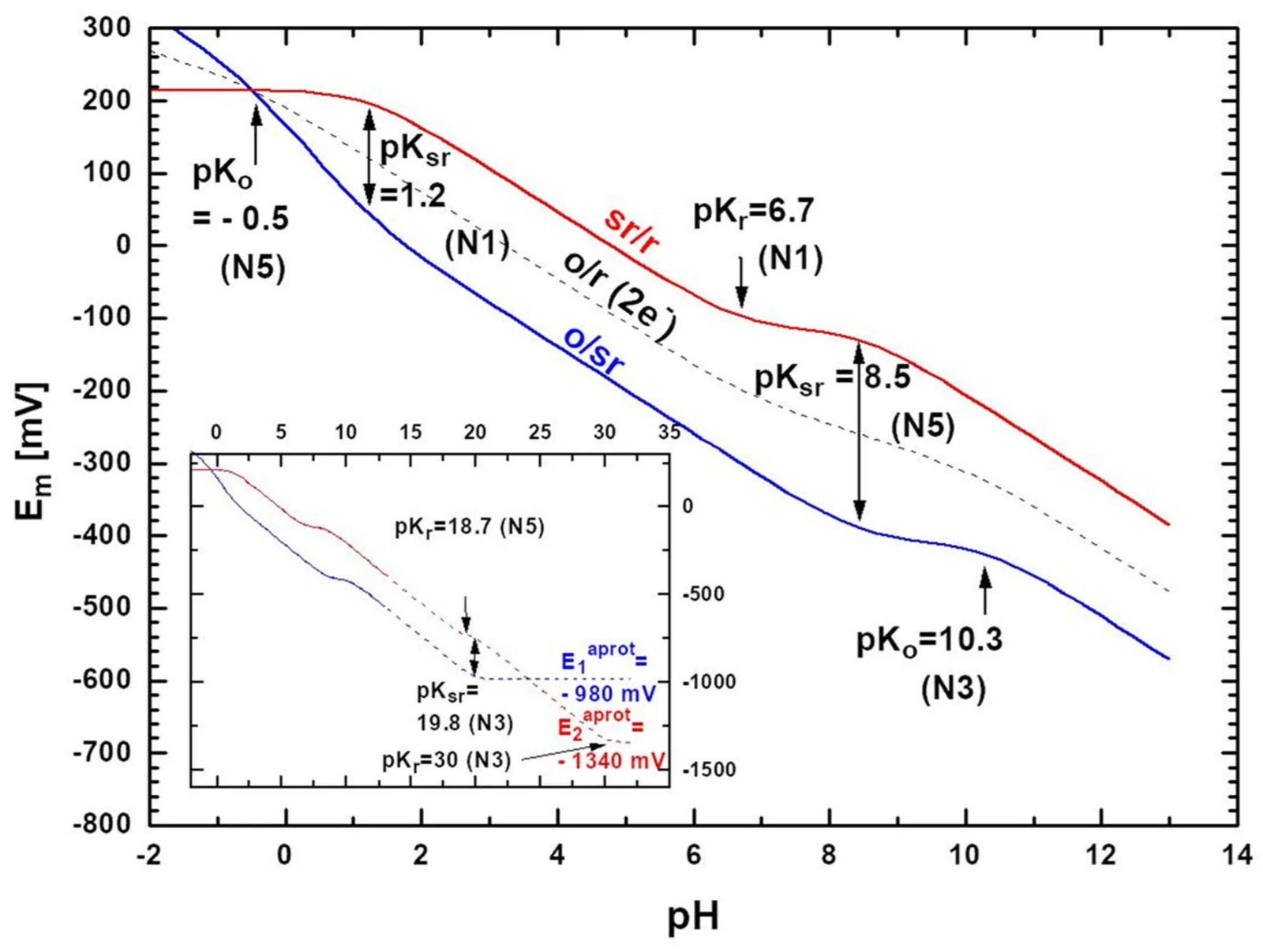
E_1,2_-vs-pH dependences in the range of pH −2 to pH 13 as determined for flavins in aqueous solution according to a synthesis of the relevant literature as discussed in the text. The main relevant pK values are indicated. The curves were generated using Formula (3), the depicted pK values and an offset to account for the out-of-range, i.e., unknown, pK values. Assuming that $E_{m}^{pH\to \infty }$ ≈ $E_{m}^{aprot}$, these remaining values were derived via adjusting Formula (3) simultaneously to the empirical curve below pH 13 and $E_{m}^{aprot}$ (as depicted in the inset).

The first exhaustive characterization of a flavin’s redox properties over a wide range of pH values dates back to a study by Michaelis and collaborators in the first half of the 20^th^ century [42,65]. Several of the redox parameters determined by Michaelis were later refined by Draper et al. [66]. These studies were performed by monitoring the optical spectrum of flavin as a function of ambient potential and pH for the oxidized, semi- (i.e., 1-electron-) reduced and fully (2-electron-) reduced states of the pigment. Only a small population of the semireduced state was observed over virtually the entire pH range examined [67,68], rendering the determination of the individual redox waves of the 1-electron transitions less accurate than that of the 2-electron transition. With the advent of electron paramagnetic resonance spectroscopy in the 1940s [69,70] specific monitoring of the radical-type semireduced state of the flavin became possible [43,44,71]. A synthesis of results from both the optical and the EPR approaches was reported in 1999 and is presently considered our best estimate of the free flavin’s redox potentials and the pH dependence thereof [15]. Figure 3 represents the E_m_/pH dependencies for the oxidized/semireduced (o/sr, blue line), for the semireduced/fully reduced (sr/r, red line) and for the 2-electron transition (o/r, dotted black line) based on the parameters reported in [15]. For representations showing both the theoretical curves and the experimental data points, see [15,65].

In the following, we will denote E_m_(o/sr) as E_1_ and E_m_(sr/r) as E_2_. It is noteworthy that this convention is inverted from that introduced by Michaelis [42] but corresponds to that frequently used for other 2-electron compounds such as quinones [72] or molybdopterins [73]. The red and blue curves shown in Figure 3 were calculated using the empirical E_m_ and pK values and the theoretical Formula (3) relating E_m_ to pH for three redox-linked protonatable groups [57]. For the o/sr- transition, K_o_ and K_r_ correspond to the empirical K_o_ and K_sr_ values while for the sr/r transition they correspond to K_sr_ and K_r_, respectively.

(3) Em=EmpH→∞−RTF∗ln[(1+aHKo3+aH2Ko2∗Ko3+aH3Ko1∗Ko2∗Ko3)(1+aHKr3+aH2Kr2∗Kr3+aH3Kr1∗Kr2∗Kr3)]

As also indicated in Figure 2 and Figure 3, only part of the total subset of pK values can be determined via the above-discussed experiments due to stability issues at very high (pH > 12) and very low (pH < −2) pH values. For protein-bound flavins (as will be discussed in Section 3.3, Section 3.4, Section 3.5, Section 3.6, Section 3.7 and Section 3.8), the stability range is even more restricted than for flavins in solution and rarely exceeds pH 4 to 10. Therefore, at first glance, the missing pK values may seem insignificant since they are not required for fitting the data points in the observable range of pHs (at least if the fitting procedure includes an adjustable constant shifting the curves to the actually measured potentials, see Section 3.9). Stabilization or destabilization of protons on N1, N3 and N5, induced by the polypeptide chain forming the binding pocket, however, is found to bring some of the “out-of-range” pKs into the observable pH region or, vice versa, moving pKs measured on free flavin out of this window. To assess the strategies of the protein environment in tuning the flavin cofactors’ redox behaviour, an at least approximate estimation of the out-of-range pKs for flavin in aqueous solutions would therefore be helpful. A rough estimation can be obtained if experimentally determined redox potentials of flavins in aprotic solvents are taken into account [74,75,76,77]. Tentatively equating these values to the $E_{m}^{pH\to \infty }$ constant in Formula (3) for both the o/sr and the sr/r transitions strongly constrains pK_sr_(N3) and indicates approximate values (±2 pH-units) for pK_r_(N5) and pK_r_(N3) as indicated in the inset to Figure 3. The out-of-range pK on the acidic side, pK_o_(N1), has been approximated by assuming a ΔpK with respect to pK_sr_(N1) of about 8 pH-units in line with several other ΔpKs seen in the scheme of Figure 2. It is noteworthy that the value of this acidic pK has no impact on the redox midpoint potentials simulated via Formula (3) in the physiologically relevant pH range and therefore does not affect the conclusions arrived at in this work.

### 3.3. Protein-Induced Stabilization of the Semireduced State

#### 3.3.1. The Flavodoxin Way

##### The Flavin-Binding Site in Flavodoxins Substantially Increases the pK Values of the N5-Proton

**Figure 4:**
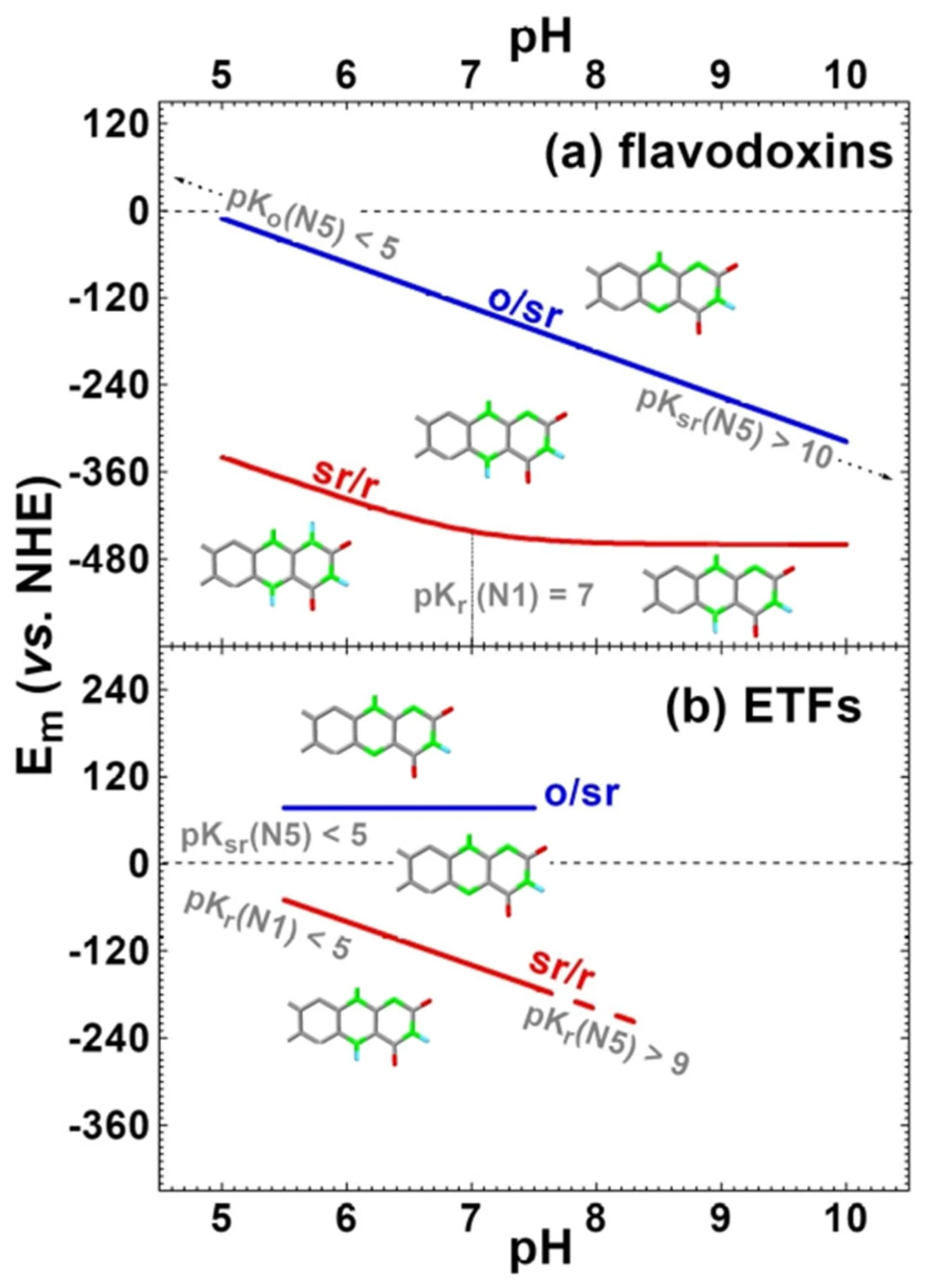
Schematic representation of the E_1,2_-vs-pH curves reported for (**a**) flavodoxins and (**b**) ETFs. Absence or presence of a proton on the protonatable nitrogen atoms of the isoalloxazine molecule is shown for all three redox states. Observed pKs or limiting values thereof are indicated.

The flavin in flavodoxins conspicuously differs from its free form in aqueous solutions (in the following denoted flavin_aq_) by undergoing two well-separated redox transitions. Flavodoxins are small electron transfer proteins and arguably are the most thoroughly studied flavoproteins both with respect to 3D structure and to physico-chemical characteristics [14,78,79,80,81,82,83,84,85,86,87,88,89]. The pH dependence of both flavin redox transitions has been measured in several flavodoxins in the range of pH 5 to about pH 9. Apart from very minor variations in pK values, all flavodoxins studied show similar behaviour as exemplified by the pH dependence of the *Desulfovibrio vulgaris* (*DesVu*) protein [83] depicted in Figure 4a (while this species has since been renamed *Nitratidesulfovibrio vulgaris*, we will refer to it by its original name used by all the relevant literature on flavodoxins). We selected the *DesVu* case from the plethora of characterized flavodoxins due to the fact that high-resolution 3D structures at all three redox states have been reported for this protein, allowing straightforward correlation of redox properties to structural idiosyncrasies.

The pattern found for the *DesVu* protein (reproduced in Figure 4a) is characteristic of the electrochemical behaviour of all studied flavodoxin-type proteins: (a) The o/sr-transition (shown in blue in Figure 4a) features a 60 mV/pH dependence and no pK value has been discerned in the analyzed range of pHs. (b) The sr/r-transition (indicated in red) displays a pK on the reduced state at around pH 7. (c) In the entire examined range, the sr-state corresponds to the neutral radical (as evidenced by the spectral features measured in UV/Vis- and EPR spectroscopy [82,90,91,92]), that is, the semireduced flavin is protonated at N5 (as identified by NMR, see above and [86]).

Taking together (a) and (c) indicates that the −60 mV/pH dependence reflects reduction-linked protonation of N5 and that N5 therefore is never protonated in the oxidized state of the flavin in the examined pH range. This implies that pK_o_(N5) must be lower than pH 5, as is also the case for flavin_aq_ (see Figure 2 and Figure 3). By contrast, pK_sr_(N5) lies above pH 10, meaning that the N5-proton of the semireduced flavin is substantially stabilized with respect to the free flavin (see Figure 2 and Figure 3). Taking furthermore observation (b) into account and considering the values for free flavin_aq_ suggests that pK_r_(N1) of the flavodoxin-bound cofactor essentially stays put (in the vicinity of 7) as compared to its free form.

The upshift of pK_sr_ of the N5-proton therefore is the only clearly discernible difference in the patterns of pK values between free flavin and the flavodoxin-bound version. The protonation states of the isoalloxazine moiety are indicated in the Pourbaix-style Figure 4a.

##### Structural Idiosyncrasies of Flavodoxins Underlying the Observed pK Changes of the N5-Proton

**Figure 5:**
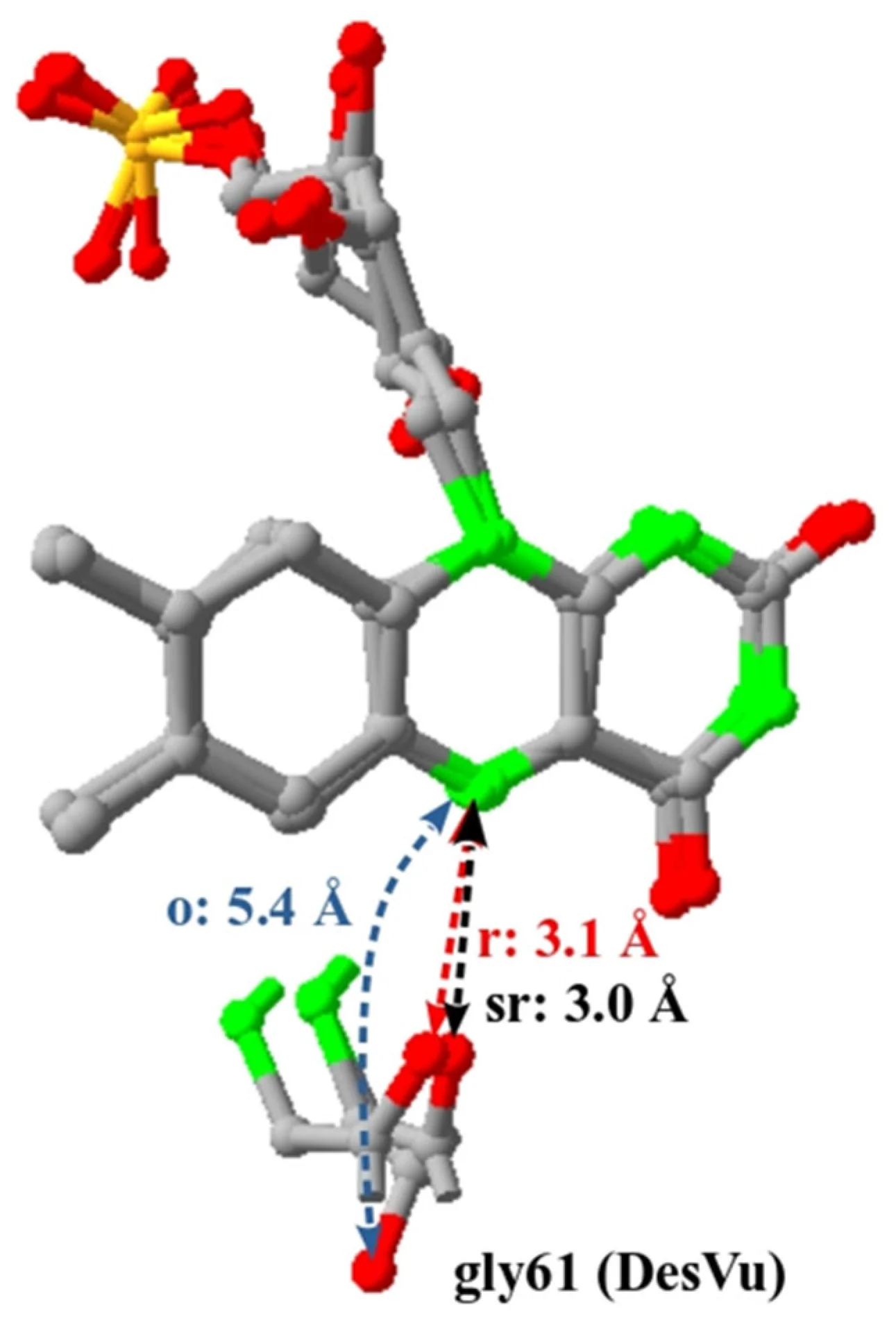
Redox state-dependent conformations of the glycine residue (Gly61) in the flavodoxin from *DesVu* and the resulting potential for a stabilizing effect of the residue’s backbone oxygen on the proton on N5 via formation of a proton-accepting hydrogen bridge. The depicted structures are based on the pdb-entries 3fx2, 4fx2 and 5fx2 for the oxidized, semireduced and fully reduced states of the flavin. 3D-rendering of these pdb-files was performed using DeepView (version 4.1.0) (http://www.expasy.org/spdbv/) (accessed on 16 October 2024).

High-resolution X-ray structures have been determined for the flavodoxin from *DesVu* in all three redox states [93]. As emphasized in the respective articles, the above-described increase in pK_sr_ of the N5-proton results from the presence of a hydrogen bond between the backbone oxygen of a glycine residue (at position 61 in the *DesVu* protein) and the proton on N5 (the H-bonding distance is indicated by a black arrow in Figure 5) of the semireduced flavin, stabilizing the protonated state of the N5-nitrogen. Since a comparable H-bond interaction is visible in the fully reduced state of the flavin (Figure 5, red arrow), the pK_r_ of N5 likely is also upshifted (although this effect cannot be observed experimentally since the pK_r_-values of both the free flavin and the flavodoxin-bound one are far above pH 10). By contrast, in the oxidized state of the *DesVu* flavodoxin, the Gly61 residue features a slightly different conformation turning its backbone oxygen away from the flavin moiety (Figure 5, blue arrow). In the oxidized state, the stabilizing H-bond interaction therefore is absent in line with the observation that no pK_o_(N5) is observed above pH 5 on the o/sr-transition (blue line) shown in Figure 4a.

The structural positioning of the loop containing Gly61 is highly conserved among flavodoxins for which 3D information is available [82,93], indicating that the above-mentioned stabilization of the N5-proton likely is a general feature of this family of proteins. The glycine itself is conserved in the overwhelming majority of cases [94].

The observed pattern of pK values in flavodoxins is therefore straightforwardly rationalized by structural features of the protein environment in the vicinity of the N5-position of the flavin. Do the above-discussed modifications in the pK of the N5-proton on their own explain the spectacular alteration in global redox behaviour, i.e., the transition from crossed-over midpoint potentials (entailing low stabilization of the sr-state) to strongly negative cooperativity (that is, uncrossed midpoint potentials and a highly stabilized sr-state) or do we have to look for additional parameters? To address this question, we have used Formula (3) to simulate the dependence of E_1,2_-vs-pH curves on the pK values of the N5-proton while maintaining all other pK values as found for flavin_aq_.

##### For High Values of pK(N5), the Flavin Cofactor Can Switch to Negative Cooperativity

**Figure 6:**
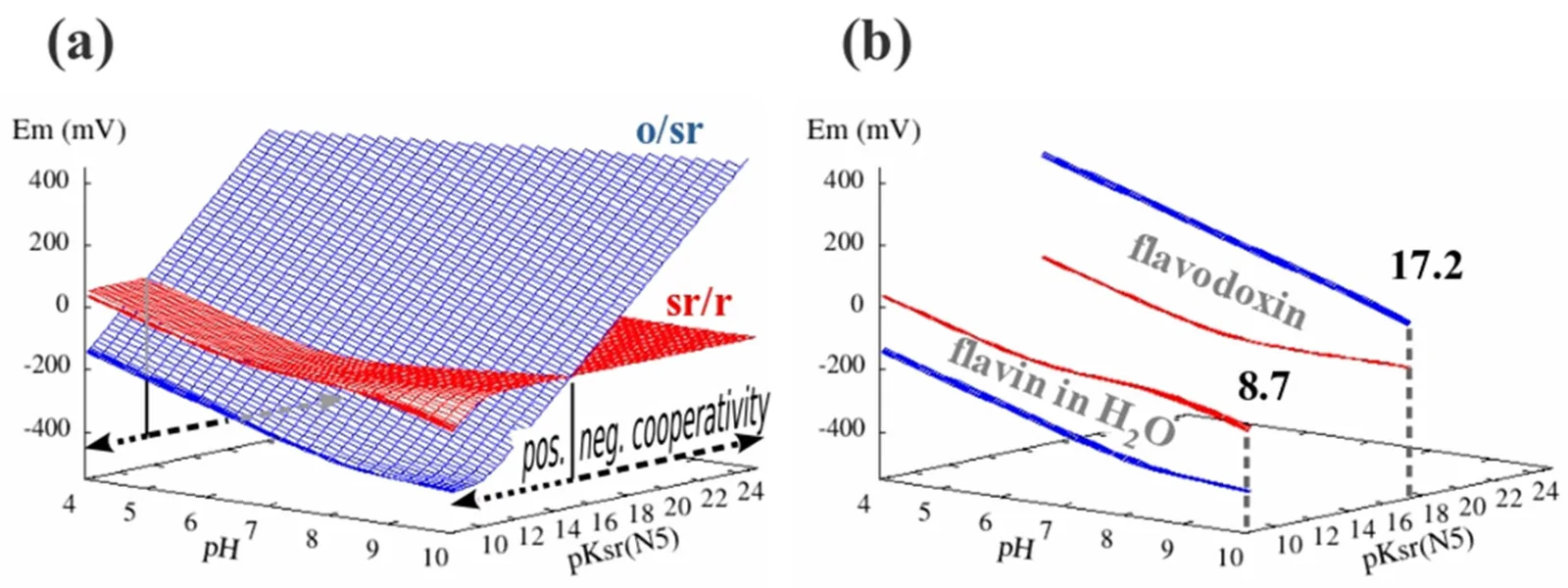
Panel (**a**) shows the E_1,2_-vs-[pH,pK_sr_(N5)]-surfaces spread out by the family of E_1,2_-vs-pH curves calculated according to Formula (3) and parametrized using pK_sr_(N5). To calculate the depicted surfaces, pK_o_(N5) was kept fixed at −0.5 as in flavin_aq_ (guided by the observation that in the oxidized state of the flavin, no protein residue is close enough to modify the pK, see Figure 5), while pK_sr_(N5) and pK_r_(N5) were allowed to vary. The interval between pK_sr_(N5) and pKr(N5) was maintained at 10.2 as in flavin_aq_. pK(N1) and pK(N3) were kept fixed to the values as in flavin_aq_. The regions of negative (E_1_ − E_2_ > 0) and positive (E_1_ − E_2_ < 0) cooperativity are indicated. Panel (**b**) extracts the individual E_1,2_-vs-pH curves corresponding to the redox cooperativity of flavin_aq_ (with a pKsr(N5) of 8.5) and to that of flavodoxins (at pK_sr_(N5) of 17.2) from the family of curves depicted in (**a**).

The theoretical pH dependences of the o/sr- and the sr/r-transitions were calculated as a function of the relevant pK values of the N5-proton using Formula (3) and as outlined in Section 3.2. Guided by the 3D information summarized above, pK_o_(N5) was kept at the same value as for free flavin while pK_sr_(N5) and pK_r_(N5) were allowed to vary with the gap between them fixed to ΔpK = 10 as suggested to be the case in flavin_aq_ (Section 3.4 and Section 3.7 deal with examples where ΔpKs may vary). The result of this simulation is shown in Figure 6a. The intersection between the two planes corresponding to the o/sr- (blue) and sr/r- (red) transitions defines the boundary between formally positive and negative redox cooperativity. The value of pK_sr_(N5) where the two planes intersect varies (smoothly) with pH and increases from 11.7 at pH 4 to 14.9 at pH 10. Above these boundary pK_sr_(N5) values the o/sr-transition becomes less reducing than the sr/r-transition and the flavin therefore enters the regime wherein the sr-state is increasingly stabilized. Furthermore, for all these pK_sr_(N5) values, the E_1_-vs-pH dependence features a slope of −60 mV/pH and that of E_2_ goes from −60 mV/pH to pH-independence above pH 7 (imposed by pK_r_(N1)). These simulated dependences perfectly mirror those obtained on flavodoxins as shown in Figure 4a and furthermore reproduce the empirical span between E_1_ and E_2_ (as shown in Figure 4a and Figure 6b) within ±5 mV for all pH values at pK_sr_(N5) = 17.2. The very satisfying correspondence between empirical pH dependences and those obtained using Formula (3) demonstrates that the general layout of the E_m_-vs-pH curves measured on flavodoxins and, in particular, the strong stabilization of the sr-form can be rationalized solely via the protein-mediated stabilization of the N5-proton.

However, as indicated by Figure 6b, the E_m_ value of the full 2-electron transition, i.e., the average of E_1_ and E_2_ is predicted by the simulation to increase (that is, to go towards more oxidizing values) while it is found to be slightly more negative in flavodoxins than it is in flavin_aq_ (cf. Figure 3 and Figure 4). The protein environment therefore obviously pushes the overall redox midpoint potentials to more negative values. Such an effect likely arises from an increasing destabilization of the cofactor-protein interaction linked to progressive reduction of the cofactor [57,95]. Indeed, in flavodoxins (as in many other flavoproteins featuring very negative midpoint potentials), the flavin cofactor shows a strong tendency to dissociate from its protein host upon full (2-electron) reduction [49,96,97,98,99,100]. The reciprocal role of pK-related and binding-affinity-induced modifications of redox midpoint potentials will be discussed in Section 3.9.

It is furthermore noteworthy that the above indicated (deduced) pK values for the transition between the regimes of positive and negative cooperativity have been obtained by approximating the measured E_1/2_-vs-pH curves by Formula (3) for a specific flavodoxin. The experimental curves, however, carry their own experimental errors, vary somewhat between flavodoxins from different species and even vary between different articles on the same species. We therefore do not claim any precision higher than ±1 unit for the boundaries between different regimes of redox cooperativity. This disclaimer also applies to all further cases analyzed below.

#### 3.3.2. The Electron-Transfer-Flavoprotein (ETF) Way

##### In Contrast to Flavodoxins, the Binding Site in ETFs Decreases the pKs of N5 and N1 of the Non-Bifurcating Flavin

The sample of characterized ETF-proteins is unfortunately much smaller than for flavodoxins and the case for generalizing the below outlined arguments to the entire family is therefore weaker than for the flavodoxin family. Nevertheless, all cases studied so far appear homogeneous with respect to 3D structures and electrochemical properties.

The ETF-family falls into two distinct classes, that is, the so-called bifurcating and the non-bifurcating enzymes [101]. Both subgroups globally feature similar 3D structures. The bifurcating representatives contain two flavin cofactors in the form of FAD, one of which displays negative 2-electron cooperativity and hence a stabilized semireduced redox state whereas the other one, the bifurcating centre, is characterized by strongly crossed-over 1-electron redox midpoint potentials and a resulting 2-electron redox transition [13]. In the non-bifurcating group, the bifurcating cofactor is replaced by an AMP molecule, the adenine-group of which occupies the same position as the corresponding moiety of FAD in the bifurcating representative [39,101]. In this section, only the non-bifurcating flavin will be considered.

As mentioned, the non-bifurcating flavin cofactor in ETFs differs from flavins in aqueous solutions by displaying strongly negative 2-electron cooperativity resulting in well-separated 1-electron redox transitions and a stabilized semireduced state of the cofactor, just as described for flavodoxins above. In stark contrast to the situation in flavodoxins, however, the semireduced state has been identified as the anionic radical [13,39,102], indicating that the FAD’s N5 remains unprotonated after 1-electron reduction. Although the redox midpoint potentials of the non-bifurcating flavin have been determined in a number of cases [48,102], to the best of our knowledge only the study by Sato et al. [13] reported the pH dependence of E_1_ and E_2_. Even in this work, the E_1,2_-vs-pH dependences were only measured between pH 6 and 7, limiting the estimation of potential pK values more severely than for the case of flavodoxins discussed above. Sato et al. found a pH-independent E_1_ and a −60 mV/pH dependence for E_2_ over the covered range of pH values (schematically represented in Figure 4b), i.e., E_1,2_-vs-pH curves substantially differing from those of flavodoxins (cf. Figure 4a). These dependences together with the finding (via UV/Vis-spectroscopy) that the semireduced state is anionic [13] show that the o/sr-transition is not accompanied by protonation of N5, requiring that for ETFs, pK_sr_(N5) must be below 5. Rather than stabilizing the proton on N5 as in flavodoxins, the protein environment of ETFs therefore appears to impede protonation of N5 as compared to flavin_aq_.

The −60 mV/pH slope of the sr/r-transition can be rationalized by two different scenarios:

(i) It arises from protonation of N5 upon reduction of the cofactor from its sr- to the r-state. This implies that pK_r_(N5) must be above 8 and that furthermore pK_r_(N1) needs to be below 6 (to avoid protonation of N1 during the sr/r-transition, which would increase the slope of the curve).

(ii) It arises from protonation of N1, requiring reverse shifts in the pK values of N1 and N5, that is, pK_r_(N5) < 6 and pK_r_(N1) > 8.

Scenario (ii) entails a substantially more pronounced downshift of the pK values of N5 than scenario (i). To decide between these two possibilities, we took advantage of the availability of high-resolution (1.45 Å) 3D information concerning the environment of the flavin in ETF [[39], pdb-entry 4L2I].

##### Structural Parameters Impacting the pK Values of the Non-Bifurcating Flavin in ETFs

**Figure 7:**
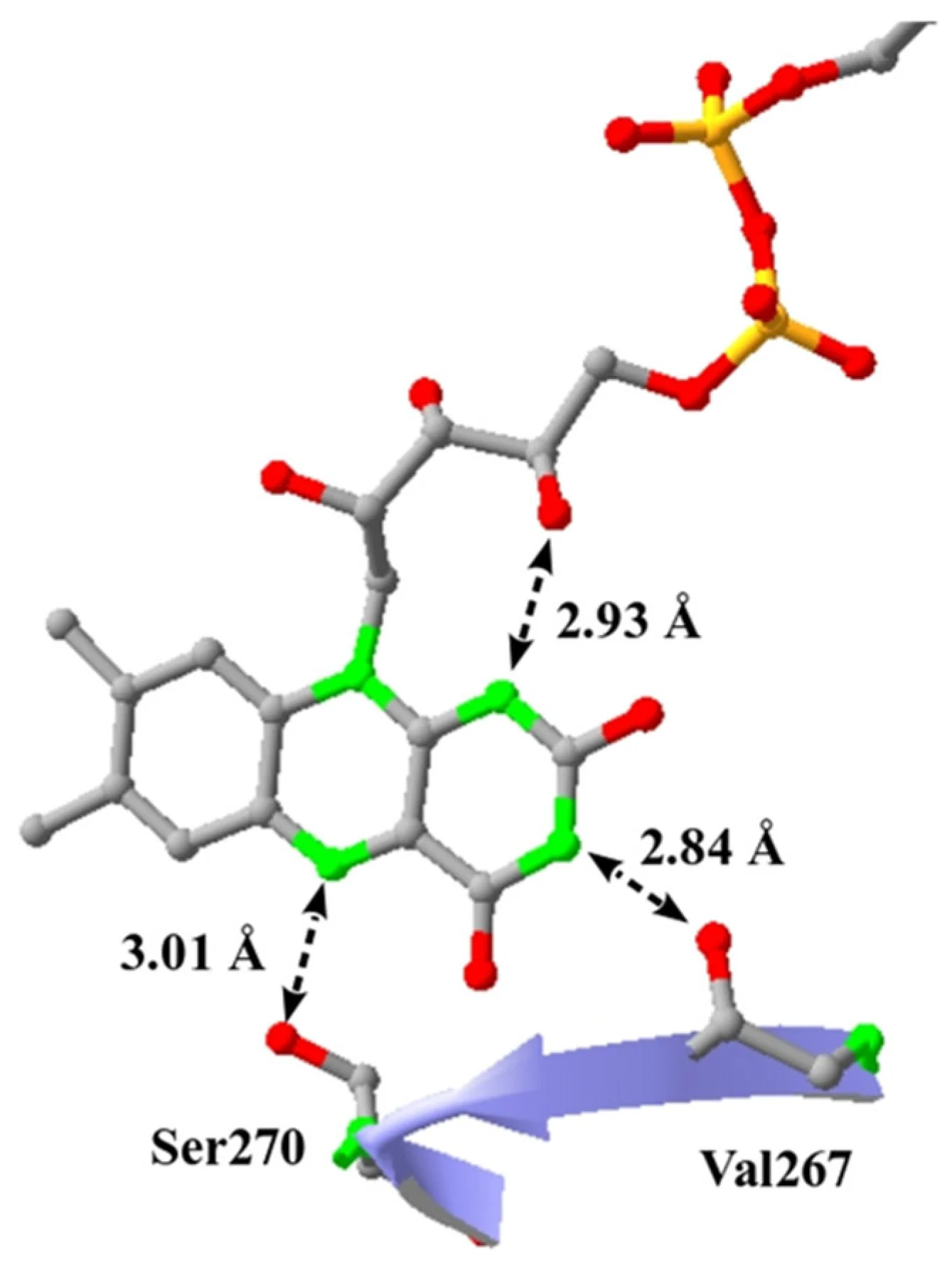
The FAD cofactor of ETF from *AciFe* and the close environment of its three protonatable nitrogens. The side-chain OH of serine 270 is likely to hamper protonation of N5 while the backbone oxygen of valine 267 will favour protonation of N3. The representation is based on pdb-entry 4L2I and is rendered using DeepView.

Figure 7 depicts features in the binding site of the flavin potentially influencing pK values of all three protonatable nitrogen atoms for ETF from *Acidaminococcus fermentans* (*AciFe*) [39]. This structure shows that the OH-group of serine 270’s side chain is turned towards N5 with an oxygen-to-N5 distance of 3Å.

Unfortunately, due to their low electron density, protons are not straightforwardly detectable in X-ray crystal structures, which therefore do not inform on whether the proton on the serine’s side-chain oxygen is turned towards or away from the N5-position of the isoalloxazine moiety. However, due to the extremely high pK of this oxygen as compared to the pK values of the N5-proton in all redox states of the flavin, we consider it very likely that the serine OH-group forms a hydrogen bridge with N5 and as a result hampers the protonation of the latter (a similar conclusion was reached by Yang and Swenson for the ETF enzyme from *Methylophilus methylotrophus* W3A1 [103]). The same reasoning applies to the likely formation of a hydrogen bridge between the proton (with an extremely high pK) on the oxygen of the FAD ribityl-group and N1 (with a hydrogen bond length of 2.9 Å, see Figure 7). By contrast, the short distance (2.8 Å) between N3 and the (necessarily deprotonated) backbone oxygen of valine 267 (Figure 7) implies that this interaction further stabilizes the proton on N3 (which already in free flavin features pK values above 10 for all three redox states). The described hydrogen-bond interactions rationalize the pK shifts assumed in scenario (i) but are at odds with a strongly increased pK_r_(N1) as required by scenario (ii).

Taken together, the above-discussed electrochemical and structural arguments support destabilizing effects on both the N1 and the N5 protons and hence a decrease in pK_r_(N1), pK_r_(N5) and pK_sr_(N5) in *Acidaminococcus fermentans*. Similar effects of the isoalloxazine’s environment on the cofactor’s pK values are likely for the whole group. The position of the amino acid interacting with N5 is occupied either by a serine (as in the case of *AciFe*) or by a threonine [104]. 3D structures of Thr-containing representatives of the enzyme family show that the O atom of the Thr (e.g., as in *Geobacter metallireducens*, pdb-entry 5OW0) is positioned exactly as that of Ser (with an O-N-distance of 3 Å). The ribityl group is also positioned in similar ways in all non-bifurcating flavins of ETFs, i.e., suitable for engaging in proton-donating hydrogen bonding with N1. The structural pattern destabilizing both the N1 and the N5 protons therefore appears to be conserved for all non-bifurcating flavins within the ETF-family.

Following the strategy applied to the case of the flavodoxins (see Section 3.3.1) we have simulated the theoretical redox properties and pH dependences thereof for the non-bifurcating flavin in ETFs by introducing the above deduced pK shifts into Formula (3).

##### Lowering Rather than Increasing pK_sr_(N5) Also Pushes the Flavin Cofactor’s Redox Behaviour into the Uncrossed Regime

**Figure 8:**
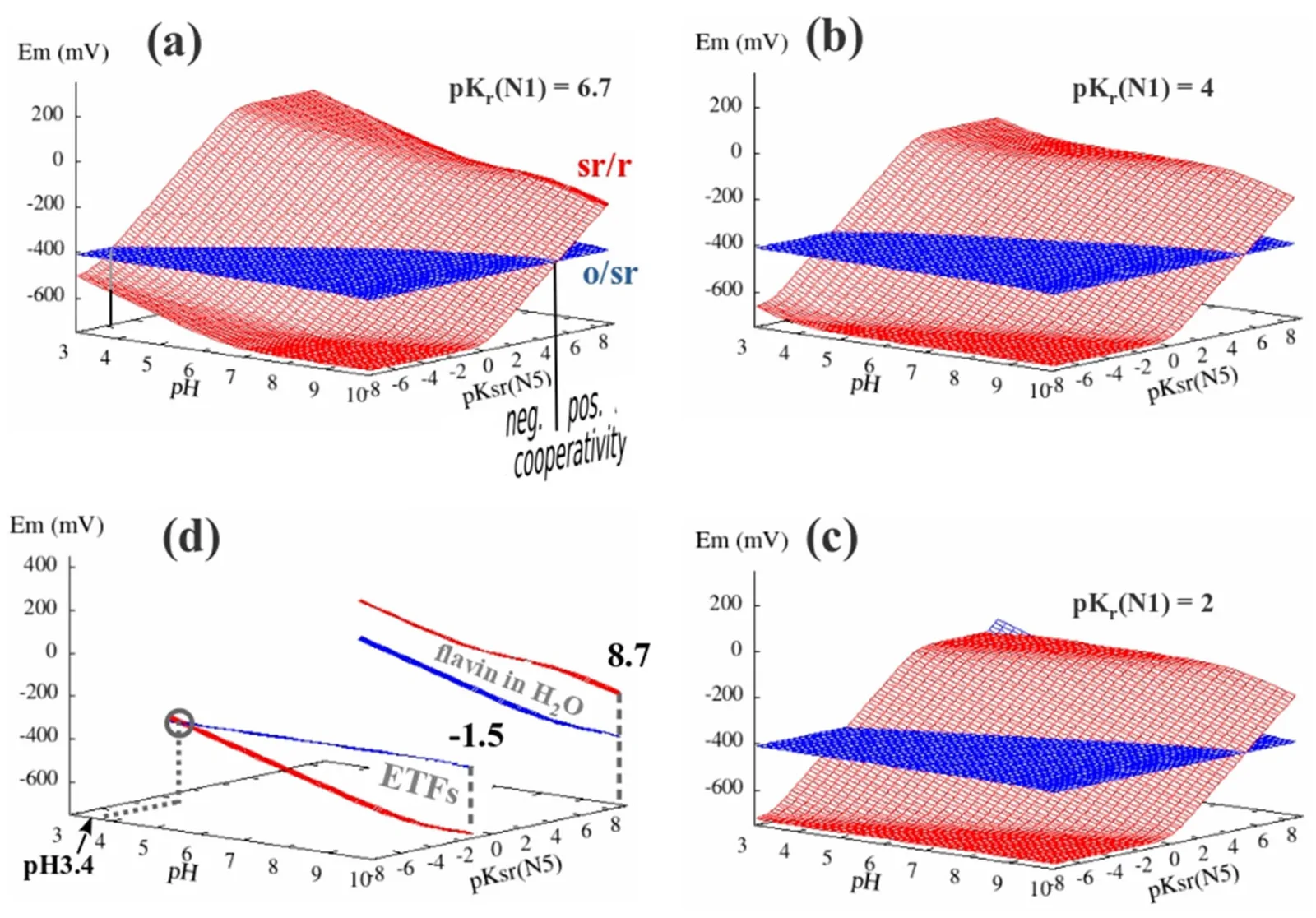
(**a**–**c**): E_1,2_-vs-[pH,pKsr (N5)] surfaces for pK_sr_ (N5) values below that of flavin_aq_. While in (**a**) the pKs of N1 and N3 were maintained as in flavin_aq_, (**b**,**c**) illustrate the effect of lowering the pK_r_ of N1 (pK_o_ and pK_sr_ of N1 were allowed to vary in parallel to pK_r_ by keeping the interval as in flavin_aq_). The family of curves shown in (**c**) contains the case matching the observed cooperativity of the non-bifurcating flavin of ETF at pK_r_(N1) = 2. This specific case is compared to that of flavin_aq_ in panel (**d**). The theoretical cross-over pH for the simulated curves furthermore is in good agreement with that suggested by the empirical data depicted in Figure 4b.

Figure 8 shows the dependence of the E_1,2_-vs-pH curves for the o/sr- and the sr/r-transitions as a function of pK_sr_(N5). The dependences were again simulated via Formula (3) and by maintaining the spacings between the three pK values of the N5-proton as found for flavin_aq_ (Figure 2 and Figure 3). In a first attempt (Figure 8a) at reproducing the ETF case, the absolute pK values of N1 and N3 were furthermore kept as in flavin_aq_ (however, see below for subsequent fine-tuning of pK_r_(N1)). Since in ETFs, the N5-proton is destabilized rather than stabilized as in flavodoxins, Figure 8a shows the respective dependences only for pK_sr_ (N5)-values below 8.7 (i.e., that of the free flavin). Below a certain value of pK_sr_(N5) (depending on pH), the two individual redox transitions switch from positive (for higher pK_sr_) to negative (for lower pK_sr_) cooperativity. For low values of pK_sr_(N5), the semireduced state therefore becomes stabilized as indeed observed for the non-bifurcating FAD in ETFs. Furthermore, the o/sr-transition (blue surface) becomes pH-independent, again mirroring the experimental data in ETFs. By contrast, for most pK_sr_(N5)-values below ~5, the sr/r-transition (red surface) shows a pH dependence steeper than −60 mV/pH, as is to be expected due to the fact that pK_r_(N1) was kept at 6.7 (as in flavin_aq_), increasing the slope below pH 7 due to the redox-coupled protonation of N1 when the FAD goes from the sr- to the r-state. Figure 8b,c show the E_1,2_-vs-pH dependences for two different degrees of destabilization of the N1-proton, that is, taking pK_r_(N1) down to 4 (Figure 8b) and to 2 (Figure 8c). In both cases, the E_2_-vs-pH slopes for pK_sr_ (N5) < 5 and between pH 4 and 8 now amount to −60 mV/pH, in line with the values observed by Sato et al. [13]. By contrast, these modifications of pK_r_(N1) do not influence the overall layout of the E_1_ and E_2_ surfaces.

Using Formula (3) again, the pK_sr_ (N5)- and pK_r_(N1)-values for which the simulated dependence most closely matches the observed situation (Figure 4b, [13]) was determined yielding pK_sr_ (N5) = −1.5 and pK_r_(N1) = 2 as indicated in Figure 8c,d. For these values, the experimental span between E_1_ and E_2_ is reproduced by the simulation within ±5 mV for all pH values and the theoretical cross-over point is found at pH 3.4, just as predicted from the empirical titration curves shown in Figure 4b. It is noteworthy that the simulated curve shows a pK_r_ at a pH of 8.5 corresponding to pK_r_(N5), outside the range covered by the experimental data on the *AciFe*-ETF.

In summary, as for the case of flavodoxins, the empirically observed E_1,2_-vs-pH curves can thus be rationalized principally by a substantial alteration of the stabilization of the proton on N5. While in flavodoxins, this proton is stabilized with respect to the situation in flavin_aq_, it is destabilized in the non-bifurcating FAD of ETFs. To account for minor details of the E_2_-vs-pH curve, a destabilization of the N1-proton must additionally be taken into account. The changes in pKs of both the N5- and the N1-proton are corroborated by structural details of the cofactor’s environment (see above).

However, again as for the case of flavodoxins, the absolute values of E_1_ and E_2_ are not correctly predicted by Formula (3) arguing for selective stabilization of the more reduced redox states of the cofactor by the binding pocket in ETF, opposite to what is required to rationalize the absolute Em values in flavodoxins.

##### A Contrasting Scenario Proposes an Alternative Rationalization for the Stabilized sr-State in *AciFe*-ETF

**Figure 9:**
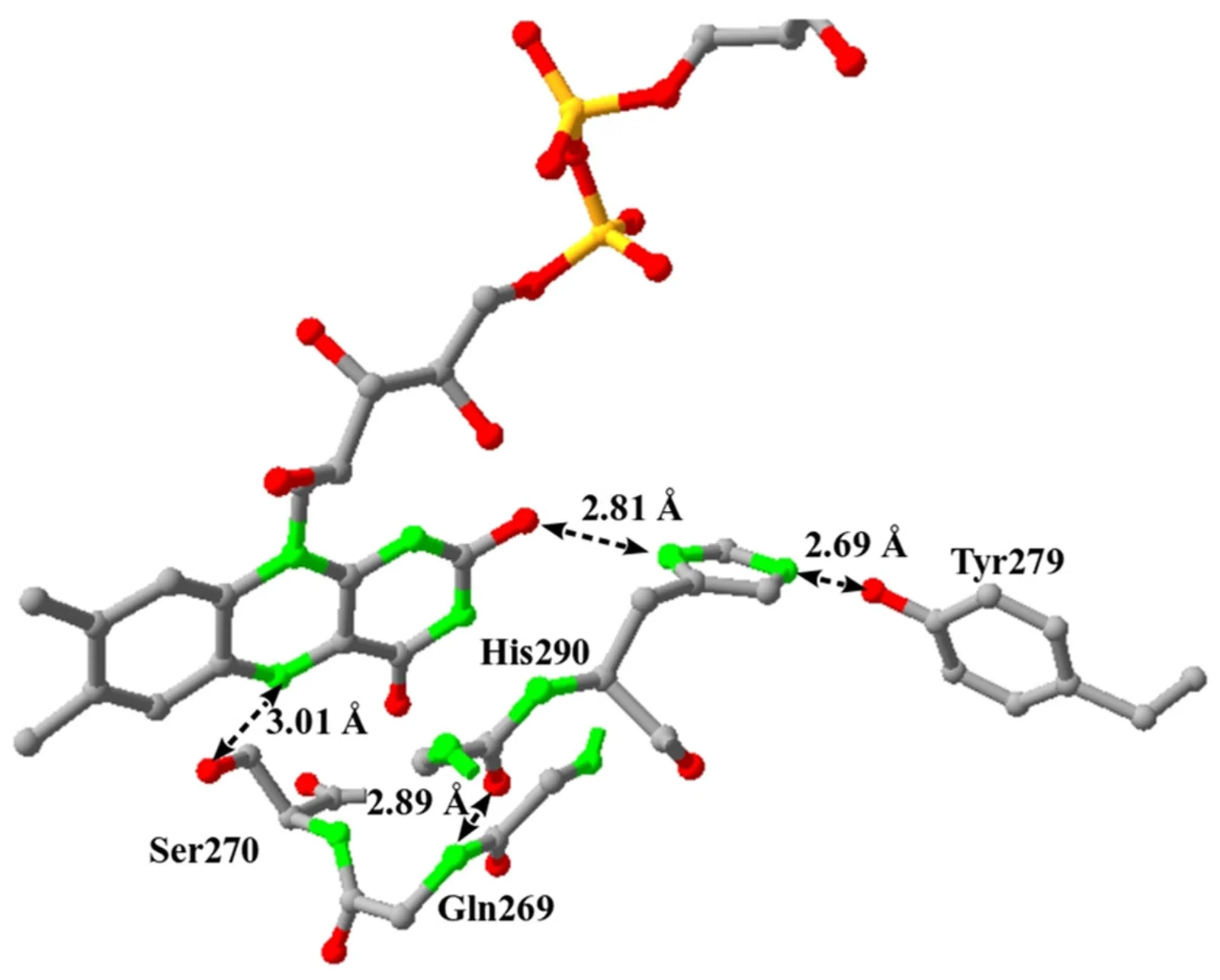
Surroundings of the flavin cofactor in ETF from *AciFe* highlighting the strong interaction between the residues proposed in [105] as influencing the flavin’s redox cooperativity and the sequence stretch harbouring serine 270, that is, the residue modulating the pK of N5 (pdb-entry 4L2I).

In 2024, Das & Miller [105] reported that site-directed variants of *AciFe*-ETF, wherein His209 was exchanged for a range of amino acids unsuited for forming proton-donating hydrogen bonds, feature strongly modified electrochemical properties and most notably a substantially destabilized sr-state of the non-bifurcating flavin. His290 engages in a strong hydrogen-bridge interaction with the O2-oxygen of the isoalloxazine moiety (Figure 9).

These effects prompted the authors to deduce that it is the presence or absence of a hydrogen bond with O2 that toggles the cofactor from positive to negative redox cooperativity. However, an inspection of the *AciFe*-structure shows that the backbone oxygen of glutamine 289, that is, the residue directly preceding His290, forms a hydrogen bridge with the backbone nitrogen of Gln269, i.e., the residue just upstream of Ser270 (Figure 9). Ser270 is precisely the residue we argue destabilizes the N5-proton and hence decreases its pK (see above). We therefore strongly suspect that mutating His290 (together with a Tyr179 fixing His290 in its specific conformation [105]) may have had an effect on the positioning of Ser270 and may hence have modified the strength of the hydrogen-bond interaction with the deprotonated N5. Unfortunately, no 3D structures of the variants reported by Das & Miller are presently available. Moreover, no pH dependences of either the wild-type or the variants have been reported so far. As mentioned above, the simulated E_1,2_-vs-pH curves feature a pK_r_ slightly above 8 due to pK_r_(N5). The determination of the pH dependences for the wild-type and the mutant may help to decide between Das & Miller’s proposal and our scenario. However, the most direct empirical tool to map the position of Ser270’s O atom would be via 3D structure determination of the above-discussed mutants.

### 3.4. Ferredoxin–NADP-Reductase: At the Boundary Between Negative and Positive Redox Cooperativity

**Figure 10:**
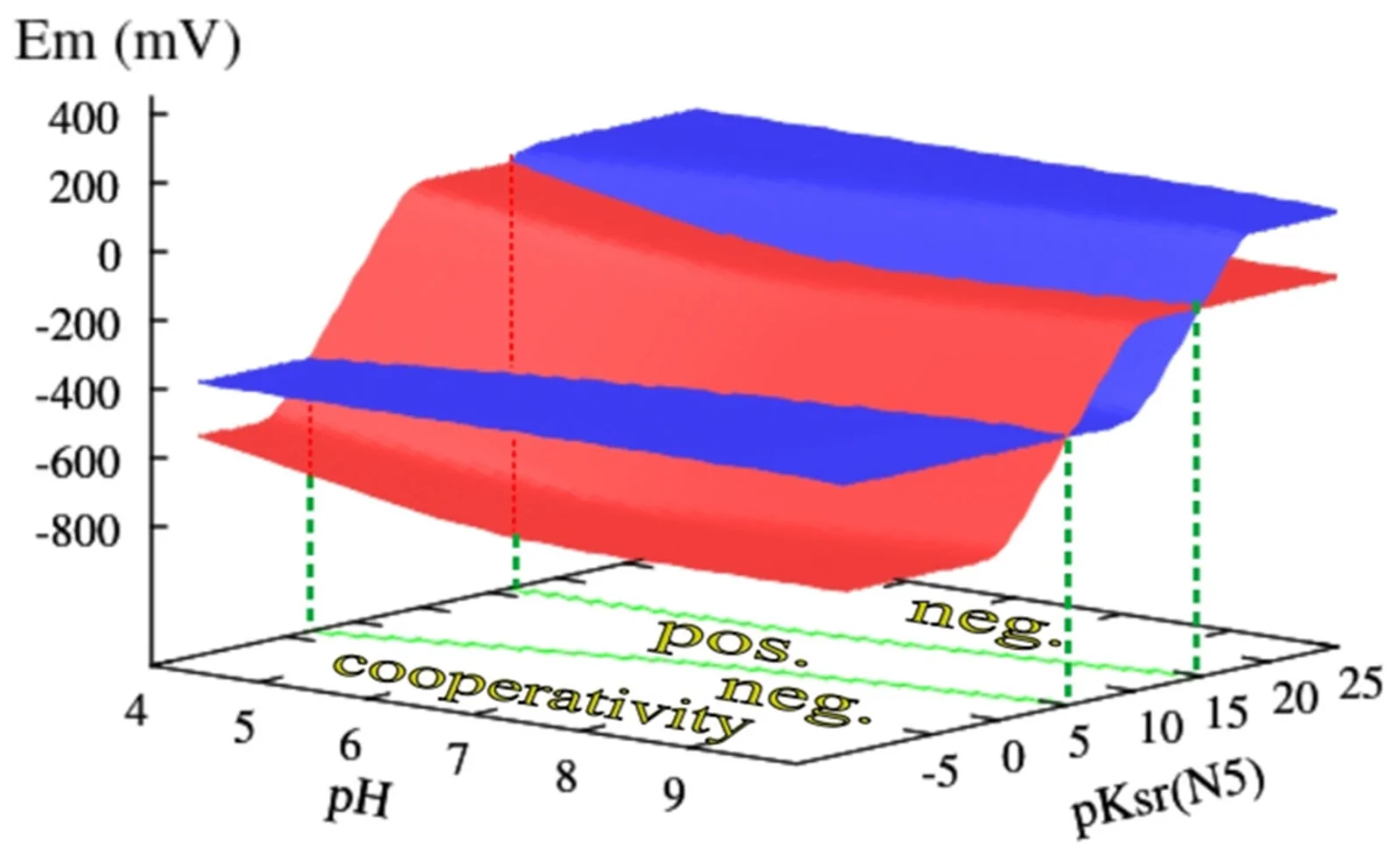
Synthesis of the situations illustrated in Figure 6 and Figure 8, encompassing pK_sr_(N5) values from −10 to +25. As demonstrated by the intersections of the E_1_ (blue) and E_2_ (red) surfaces, the cofactor passes from negative cooperativity at low pK_sr_(N5) into an intermediate regime of positive cooperativity and then back into negative cooperativity at high pK_sr_(N5). The respective spreads of these differing regimes are obtained by projecting the intersection curves onto the base plane of the coordinate system. All three pKs of N5 were allowed to vary in the simulation shown with intervals as observed for flavin_aq_.

As emphasized previously [22], the regimes of negative and positive redox cooperativity form a continuum. Figure 10 shows that tuning pK_sr_(N5) through the range of −5 to 20 steers the flavin cofactor from negative cooperativity (with an anionic sr-state) through a regime of positive cooperativity (with a destabilized sr-state) and then back again into negative one (now featuring a neutral sr-state).

In biological examples, this allows flavoenzymes to catalyze 1-electron reactions as discussed above for flavodoxins and non-bifurcating ETFs, exclusive 2-electron transfer reactions as in the enzymes Ndh-2 or SQR (see below, Section 3.5), but also to serve as gating agents mediating between 1-electron and 2-electron redox partners (see Figure 1 in [22]). Empirically, such “gating flavin cofactors” are seen to tune their individual 1-electron transitions to feature roughly similar redox midpoint potentials [22]. Incidentally, this also appears to be true for quinone-based systems [106].

**Figure 11:**
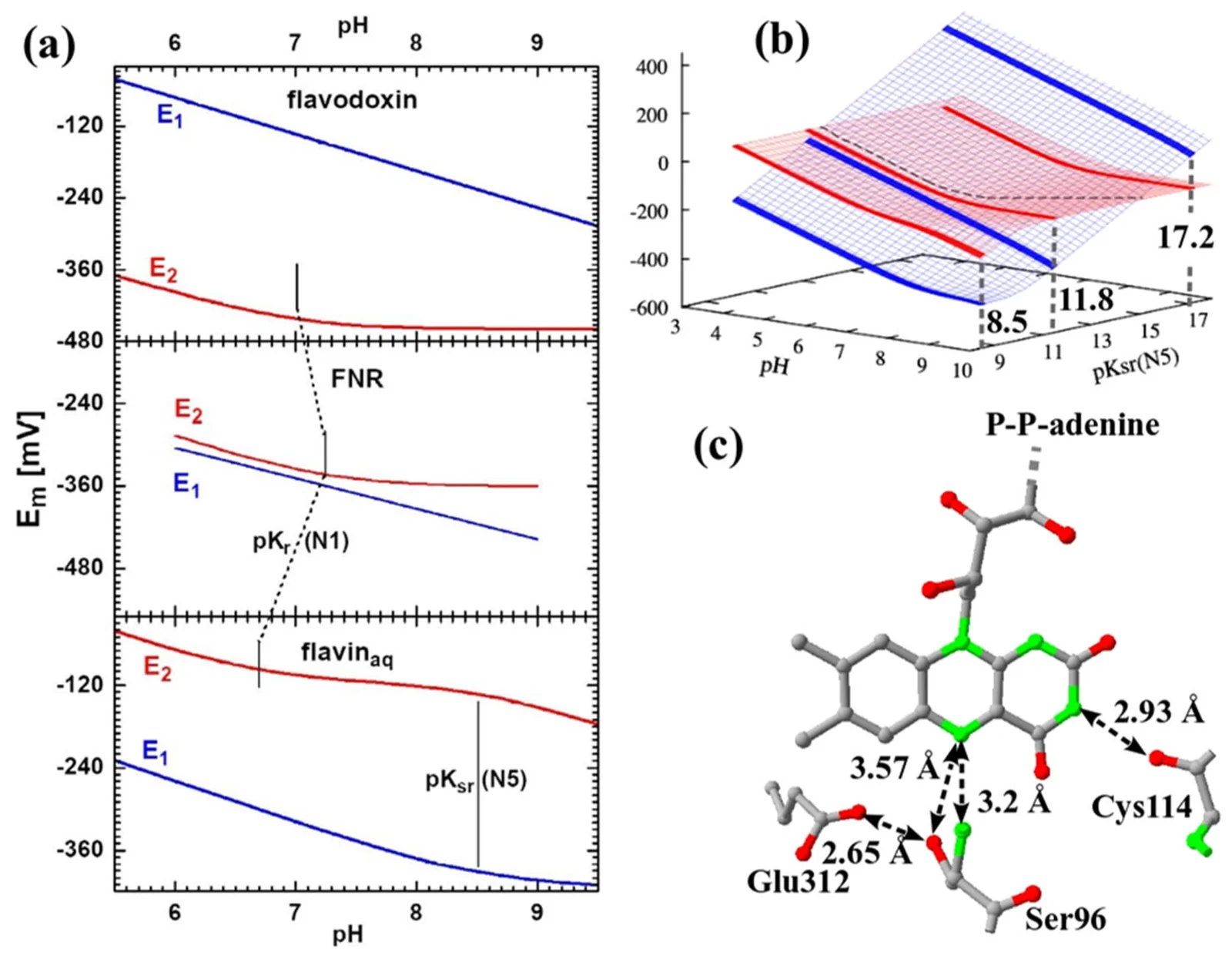
(**a**) Comparison of the empirical E_1,2_-vs-pH curves of flavin_aq_ (**bottom panel**), flavodoxin (**top panel**) and FNR (**middle panel**) highlighting the fact that FNR shows redox behaviour close to the transition from negative (e.g., in flavodoxins) to positive cooperativity (as in flavin_aq_). (**b**) This plot conveys the fact that solely varying N5’s pK values allows the reproduction of the experimental signs and degrees of redox cooperativity for cases as different as ETF, FNR and flavodoxin (and, of course, flavin_aq_ as shown in Figure 6 and Figure 8, which, however, was omitted in this figure for the sake of clarity). For a satisfying representation of the flavodoxin case, pK_o_(N5) was fixed as in Figure 6, which has a negligible impact on the shape of the surfaces for pK_sr_(N5) below 15. All other pK values are as determined for flavin_aq_. The dotted line denotes the intersection between the E_1_ and E_2_ planes. (**c**) 3D structure of the surroundings of the flavin’s protonatable nitrogens in FNR (pdb entry 1FNB).

The increasing difficulties in detecting the (substoichiometrically populated) sr-state entails an unfortunate paucity of available data sets on the redox behaviour of gating flavoenzymes. A notable exception is provided by the enzyme Ferredoxin–NADP-Reductase (FNR). Corrado et al. [107] have succeeded in measuring the pH dependence (in the range of pH 6 to pH 9) of the individual 1-electron transitions of FNR via detection of the characteristic spectral bands of the neutral semireduced flavin in UV/Vis spectroscopy. Figure 11a represents these E_1,2_-vs-pH curves (middle panel) in comparison to those of flavodoxins (top panel) as well as to those of flavin_aq_ (bottom panel) in the same pH-window. E_1_ and E_2_ are indeed found to occur at almost the same potential in FNR resulting in the presence of the neutral sr-state at this potential in only roughly 1/3 of the centres (see Figure 4b as well as the Supplementary Materials in [38]). While the stabilization of the sr-state obviously differs substantially between the three systems represented in Figure 11a, the overall shapes of the E_1,2_-vs-pH dependences are strikingly close. A pK on the r-state in the vicinity of 7 (attributed to the N1-proton) is a common feature of all three E_2_-vs-pH curves. As mentioned above, the UV/Vis spectra demonstrate that the sr-state in FNR is neutral suggesting that pK_o_(N5) must be below 5. In contrast to free flavin but similar to the situation observed in flavodoxins, neither the E_1_-vs-pH nor the E_2_-vs-pH curve in FNR show the pK of 8.5 attributed to the N5-proton in the semireduced state, suggesting that, just as in flavodoxins, this pK is upshifted to values above 9.

The observed features of the E_1,2_-vs-pH curves together with the neutrality of the sr-state therefore indicate that modification of pK(N5) is the dominant parameter distinguishing the redox pattern of FNR from those of free flavin and flavodoxin. Hence, the family of curves resulting from the variation of pK_sr_(N5), as depicted in Figure 6a and Figure 10, can be expected to encompass the redox pattern displayed by FNR. Indeed, using a pK_sr_(N5) of 11.8 in Formula (3) nicely reproduces FNR’s redox behaviour (Figure 11b). As for flavodoxins, the absolute values of E_1_ and E_2_ are more negative than the simulated curves, again arguing for stronger affinity of the flavin to its binding pocket in the oxidized state as compared to the more reduced redox states (see Section 3.9).

Conveniently, 1.7 Å X-ray structure of this enzyme (pdb-entry 1FNB) is available [108]. The protein residues close to the protonatable nitrogens of the flavin are represented in Figure 11c. While N3 engages in a proton-donating H-bridge with the backbone oxygen of Cys114 (at a distance of 2.93 Å), no potentially interacting residues or water molecules are present closer than 3.4 Å from N1. As mentioned above, the further stabilization of the N3-proton does not influence the redox behaviour in the relevant pH-window due to the high value of N3’s pK values at all redox states of the flavin.

A conserved serine residue (Ser96) in the vicinity of N5 is frequently discussed as being crucial for FNR’s functional properties [51,109,110]. The side-chain oxygen of this residue is never closer than 3.5 Å to N5 in all reported 3D structures, that is, unlikely to engage in hydrogen-bond interactions with N5. Furthermore, the proton on Ser96 likely faces away from the flavin to form a strong (2.65 Å) H-bridge with Glu312 (Figure 11c) [111]. We therefore consider Ser96’s side-chain OH-group as unsuited for influencing the pK values of N5. By contrast, the α-amino nitrogen of the same serine residue is positioned at 3.2 Å from N5 and the stereochemistry of the α-amino nitrogen imposed by the backbone trajectory results in its lone-pair orbital pointing almost directly towards N5. The pK value of the proton on the backbone α-amino nitrogen in peptides has been determined to be in the range of 7 to 8 [112]. Considering that the pK of the N5-proton in oxidized, semireduced and reduced flavin_aq_ is at −0.5, 8.5 and around 18, respectively, it is likely that in the o-state of FNR’s flavin cofactor electrostatic and steric interactions between the α-amino protonation site and that on N5 will result in further destabilization of the proton on N5 (hence inducing a lower pK value) and a stabilization of the α-amino proton (entailing an increase in its pK value). By contrast, in the sr- and the r-state, the reverse pattern will prevail, that is, the N5-proton will become slightly stabilized by the deprotonated α-amino nitrogen. This scheme will persist through the entire range of circumneutral pH values covered by Corrado et al.’s redox titrations. Similar patterns have been observed in other redox enzymes [113,114,115] and are referred to as “rocking-proton” situations [113].

As explained above, the rocking-proton situation in FNR will result in a lowering of pK_o_(N5) and an increase in pK_sr_(N5) and pK_r_(N5) implying a widening of the ΔpK-gap between the oxidized state on one side and the sr- and r-states on the other side as compared to flavin_aq_. Since the pK_o_(N5) therefore is expected to be far below zero, it does not influence the E_1,2_-vs-pH curves in the empirically accessible region of pH values. From Formula (3), a pK_sr_(N5) of 11.8 (corresponding to a weak stabilization of the N5-proton) and a ΔpK(N5) = pK_r_(N5) − pK_sr_(N5) similar to that in flavin_aq_ are predicted (Figure 11b) to yield the type of redox cooperativity as observed in FNR (Figure 11a, middle panel).

### 3.5. Onwards to Flavoenzymes Featuring Destabilized sr-States: The Cases of Ndh-2 and Sulfide Quinone Reductase

Further destabilizing sr-states in flavoenzymes obviously compounds the experimental difficulties in determining the redox midpoint potentials of the individual 1-electron redox transitions, let alone their pH dependences. While positive cooperativity grows, progressively more pronounced 2-electron Nernst curves together with decreasing spectral contributions of the sr-state are observed [42,65]. The above developed analytical approach will in the following be extended to the cases for which the required redox and structural information is available.

More pronounced positive cooperativity (that is, increasingly negative ΔE) will further destabilize the semireduced state of the flavin and thus progressively favour direct 2-electron transfer. Such low to moderate positive cooperativities roughly correspond to the redox properties exhibited by flavin_aq_. Indeed, in available 3D structures of such enzymes, the protein environment of the cofactor does not engage in interactions with the protonatable nitrogens of the flavin moieties and therefore is unlikely to modify the flavin’s pK values and thus the relative position of the two 1-electron transitions. In Ndh-2, for example, no protein-derived atom is closer than 3.8 Å to the N5-proton, the N3 proton is in proximity only to a water molecule (just as is the solvent exposed free flavin) while the N1 proton might be very slightly destabilized by the presence of a backbone nitrogen. These patterns are observed in the two analyzed representatives of the Ndh-2 family (i.e., the enzyme from *Mycolicibacterium smegmatis*, pdb-entry 9MQY, and that from *Caldalkalibacillus thermarum*, pdb-entry 5KMS) and a similar pattern is present in sulfide-quinone-oxidoreductase, where both N3 and N5 protons are only in proximity of water molecules and the N1 proton appears slightly destabilized by a ribityl-OH proton (pdb-entry 3HYW from *Aquifex aeolicus*).

### 3.6. The pK-Dependent Redox Landscape of the Flavin Cofactor

As evidenced by the cases of flavodoxins, ETFs and FNR, the protein environment modifies the pK values of the three protonatable nitrogens of the isoalloxazine moiety inducing substantial alterations of the E_1,2_-vs-pH curves of the o/sr- and the sr/r-transitions. As is obvious from Formula (3), the characteristics of the o/sr-transition are affected by the pK_o_ and pK_sr_ values of the protonatable nitrogens while the sr/r-transition is controlled by their pK_sr_ and pK_r_ values. The two individual 1-electron redox transitions thus can vary in largely independent ways allowing to steer the cofactor into redox regimes featuring either positive or negative redox cooperativity resulting in more or less stabilized sr-states. This phenomenon is illustrated in Figure 10 with respect to the impact of pK(N5)-modifications and in Figure S1 additionally depicting for example the effect of pK(N1).

These figures therefore illustrate that the pKs of all three protonatable nitrogens potentially influence the redox cooperativity of the flavin’s full 2-electron reduction, resulting in a complicated, multifactorial, relationship between the set of relevant pK values and the sign and strength of redox cooperativity of the flavin cofactor. To provide a more synoptic and comprehensive picture of this relationship, we will in the following try and represent the influence of all three protonatable sites on the sign and extent of redox cooperativity. To this end, we will use a 3D-coordinate system with pK_r_(N1), pK_sr_(N5) and pK_o_(N3) as x, y and z axes. In a first approximation (however, see Section 3.7), we assume the same interval between pKs in the three redox states as in flavin_aq_ for calculating E_1_ and E_2_ via Formula (3). A pH of 7 is used for these evaluations of Formula (3) since the vast majority of flavoproteins are intracellular and will therefore experience pH values in close vicinity of neutrality. Each point in the [pK_r_(N1),pK_sr_(N5),pK_o_(N3)]-space is identified (via heatmap-type colour-coding) by its (E_1_ − E_2_) value, that is, its specific redox cooperativity.

**Figure 12:**
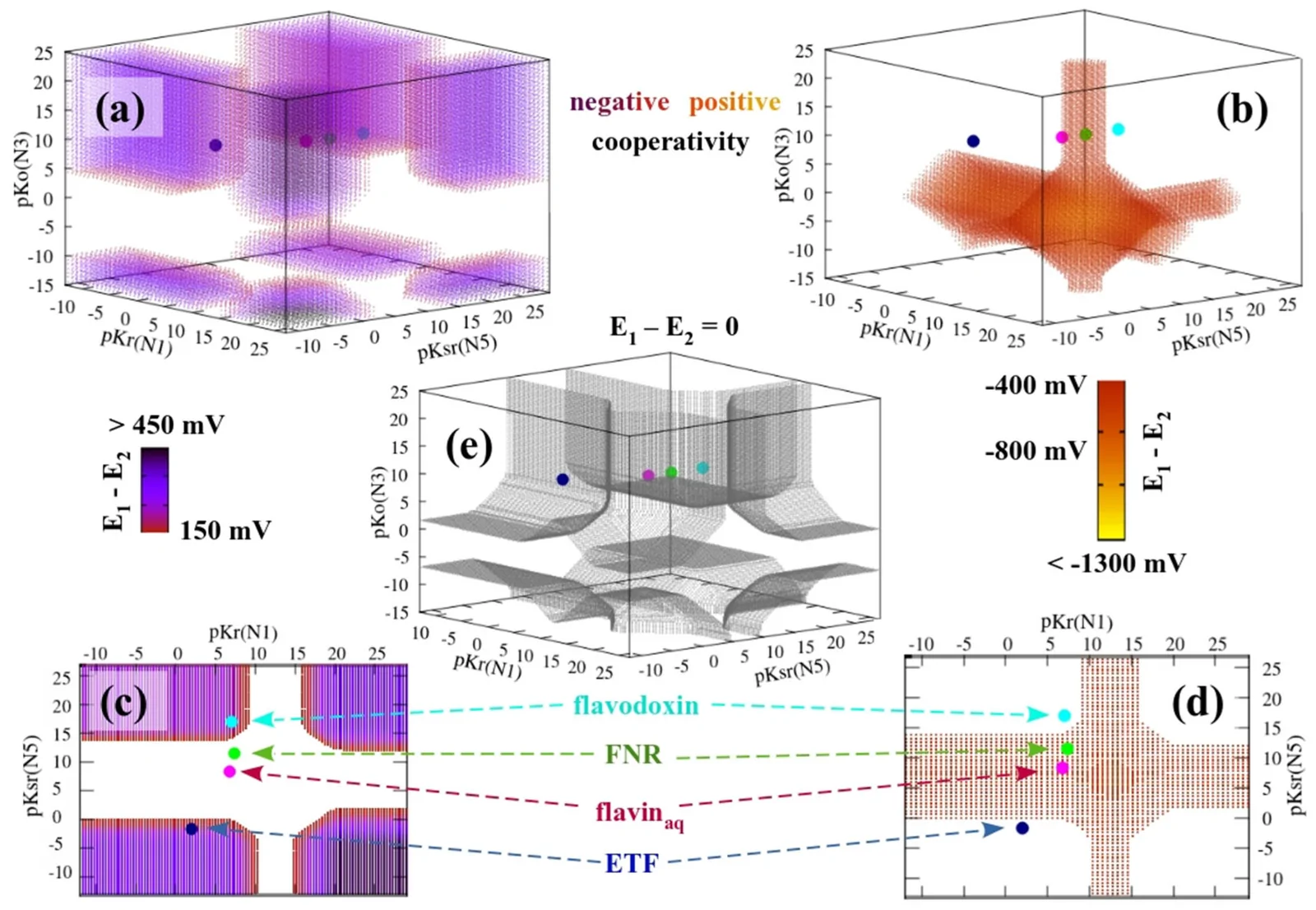
Voxelized 3D representation of the volumes in [pK_r_(N1),pK_sr_(N5),pK_o_(N3)]-space of regions of negative (E_1_ − E_2_ > 450 mV, (**a**)) and positive (E_1_ − E_2_ < −400 mV, (**b**)) redox cooperativity. Extents of negative and positive cooperativity are colour-coded in blue-to-violet or red-to-yellow), respectively (see heatmaps below the 3D plots). The lower panels (**c**,**d**) represent top views onto the 3D-coordinate systems (i.e., collapsing the pK_o_(N3)-axis). Panel (**e**) (in the centre of the figure) represents the boundary between negative and positive redox cooperativity (E_1_ − E_2_ = 0 mV). The respective positions of the empirically analyzed cases (flavin_aq_, flavodoxin, ETF and FNR) are indicated as coloured dots. For a better 3D apprehension of the volume characterizing positive cooperativity, see the animated Figure S2 in the Supplementary Materials.

Figure 12 shows voxelized representations of the volumes in this [pK(N1),pK(N5),pK(N3)]-space where the flavin undergoes either 2-electron transitions with negative cooperativity (Figure 12a,c, colours from orange to dark blue denoting increasing stabilizisation of the sr-state) or positive one (right side of Figure 12b,d, with orange to yellow standing for increasingly destabilized sr-states). The plot in the centre of Figure 12 features the surfaces delimiting volumes of positive and negative cooperativity (i.e., corresponding to ΔE = E_1_ − E_2_ = 0). The positions of flavinaq (magenta) as well as of the cofactors in flavodoxin (cyan), ETF (dark blue) and FNR (green) are also specified in all 3D plots shown in Figure 12.

Figure 12 illustrates that negative cooperativity is brought about by sets of points in the [pK(N1),pK(N5),pK(N3)]-space mapping to discontinuous volumes. This rationalizes the observation that quite similar ΔE = E_1_ − E_2_ values are found (for a given pH) in flavodoxins and the non-bifurcating flavin in ETFs despite their strongly dissimilar set of pK values positioning the two cases into topologically disconnected volumes of negative cooperativity (Figure 12a,c). By contrast, the volume in [pK(N1),pK(N5),pK(N3)]-space defining positive cooperativity (Figure 12b,d) corresponds to a topologically continuous 3-dimensional manifold. In the “core” of this topological manifold, that is, at pK_r_(N1) ≈ 12.5, pK_sr_(N5) ≈ 7 and pK_o_(N3) ≈ −3, ΔE values slightly lower than −800 mV are predicted by Formula (3) (orange voxels). Under the parameters (ΔpK values) used to generate Figure 12, such low values are only attained in this “core”-region but not in the “arms” of the topological volume representing positive cooperativity.

### 3.7. Electron Bifurcation in Specific Bi/Confurcating Flavoenzymes Goes Hand in Hand with a Structural Motif Inducing Extreme Positive Redox Cooperativity

By analogy with the better studied quinone-based enzymes [22,24,25] and in contrast to the enzymes mediating 1-to-2-electron gating or exclusive 2-electron transfer reactions, substantially more extremely positive cooperativities are likely to also be needed for solidly coupling the exergonic and the endergonic half-reactions in bi/confurcating flavoenzymes. Estimations guided by results obtained on quinone-based bi/confurcation call for ΔE values lower than −800 mV [25]. Correspondingly, the ΔE for the bifurcating flavin in the ETF enzyme from *Clostridium difficile* has been proposed to be close to −900 mV [116]. As mentioned above, truly empirical values for ΔE = E_1_ − E_2_ values are notoriously hard to come by. To our knowledge, only a single semi-direct measurement of ΔE was performed on bi/confurcating flavoenzymes via monitoring electron transfer kinetics in the enzyme Nfn [27]. These latter data suggest ΔE values in the range of −1200 to −1300 mV. However, in the simulations shown in Figure 12, no ΔE values below −850 mV were observed. Furthermore, these very low ΔE values are only reached in the core region of the volume depicted in Figure 12b,c. To move the redox behaviour of flavin_aq_ into this core region, the proton on N1 must be stabilized corresponding to an increase in pK(N1) by 5 units while that on N3, by contrast, needs to be substantially destabilized yielding pK(N3) downshifts of more than 10 units (see Figure 12). In particular with respect to the protonatable site on N3, the vast majority of 3D structures of enzymes containing bifurcating flavins indicate stabilization rather than strong destabilization of the N3 proton. How then may those flavoenzymes which perform strongly coupled bi/confurcating reactions attain the required low values?

**Figure 13:**
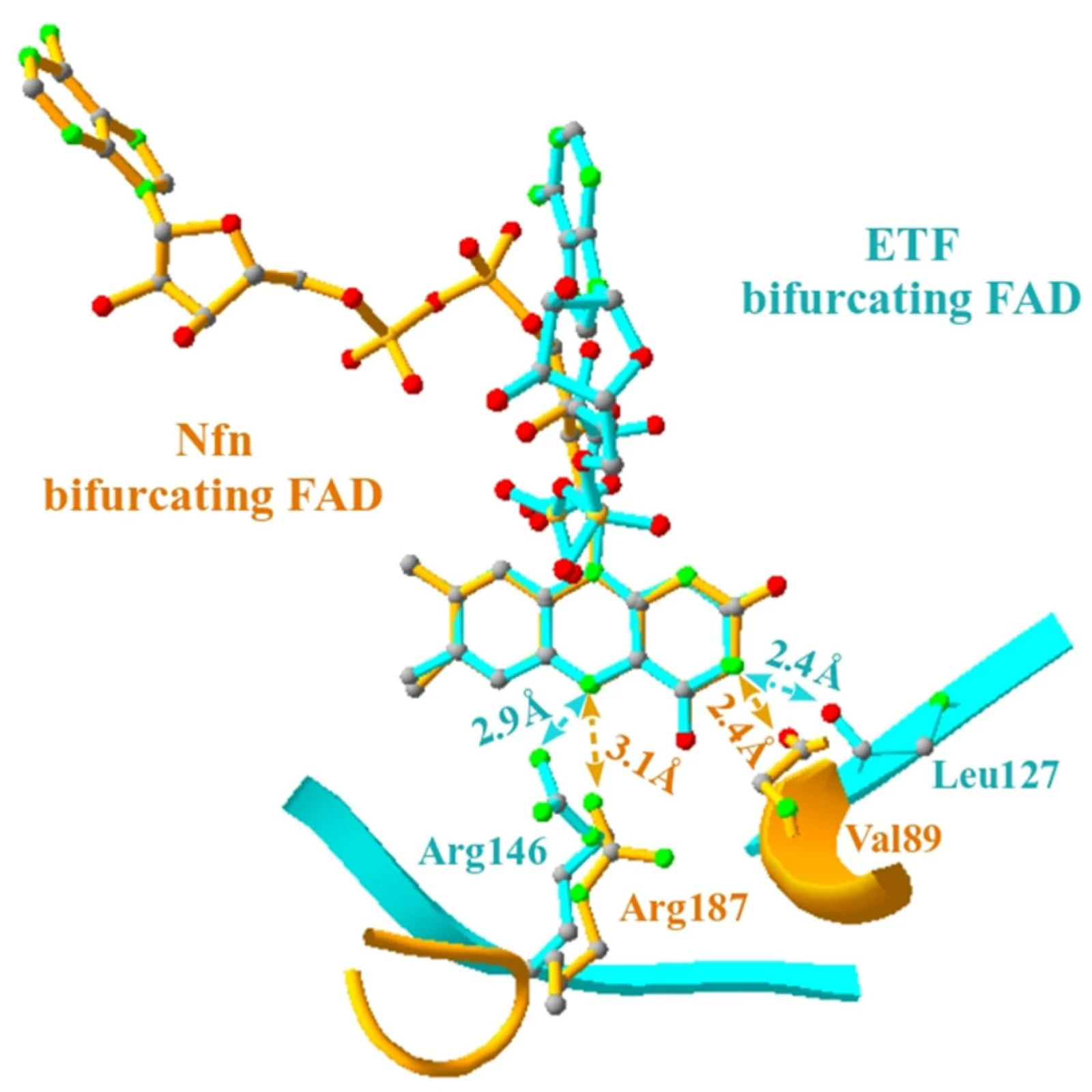
Structural superposition of the isoalloxazine moieties of the bifurcating flavin in ETF (bonds and secondary structures in blue) and of the bifurcating flavin in Nfn (bonds and secondary structures in orange) together with the close environments of their protonatable nitrogens. DeepView rendering used the pdb-entries 4L2I for ETF and 5JCA for Nfn.

**Figure 14:**
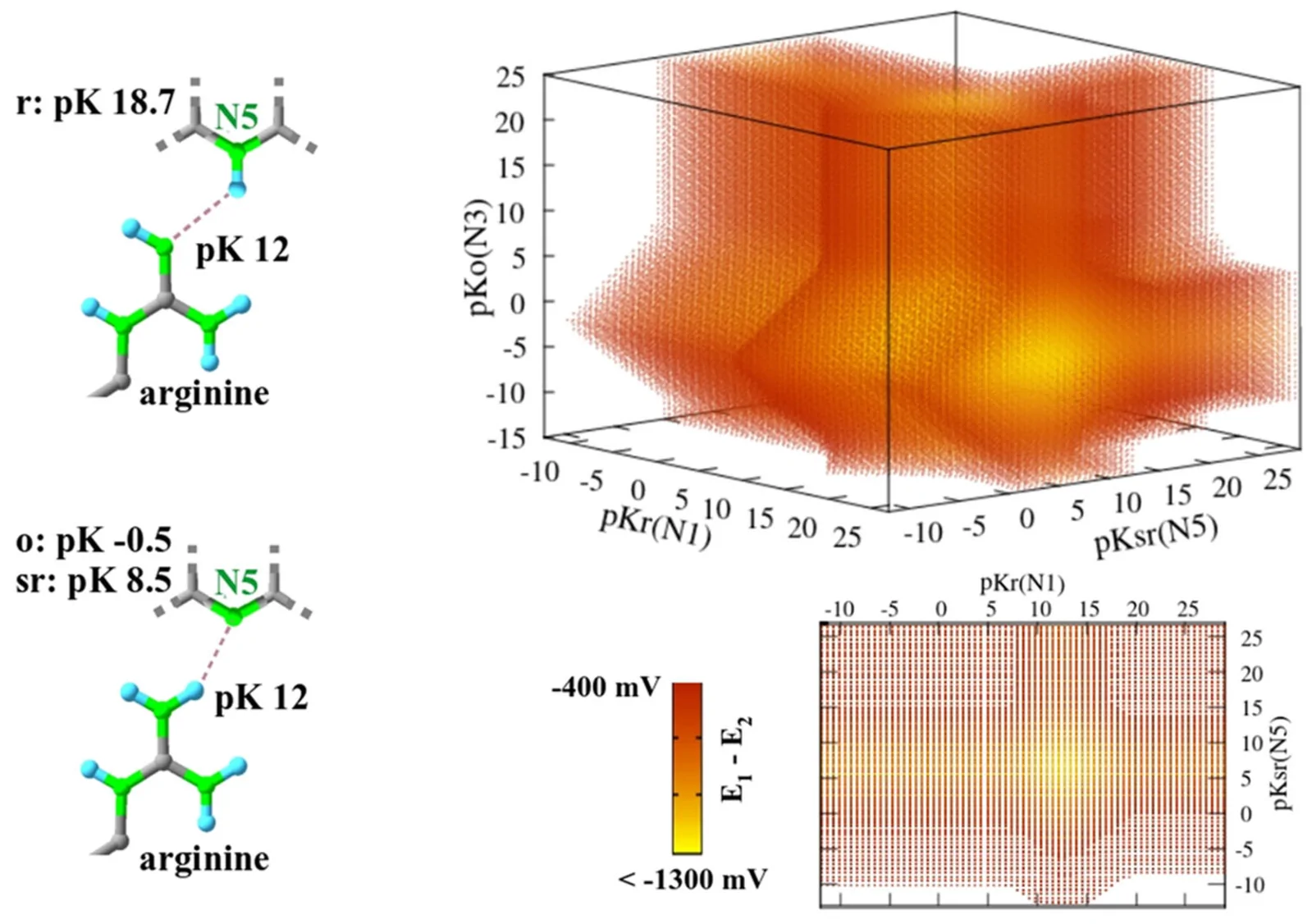
The left side shows the swivelling of the hydrogen bond between N5 and the closeby arginine residue as a function of the isoalloxazine’s redox state. While in the o- and sr-states, N5 will engage in a proton-accepting interaction (hampering its own protonation); it will serve as the proton-donor in the r-state (pushing its pK_r_ to higher values). The right side voxel representation (as in Figure 11) shows the effect of increasing the interval between pK_o_(N5) and pK_sr_(N5) on one side and pK_r_(N5) on the other. The volume of the regime of positive redox cooperativity (E_1_ − E_2_ < −400 mV) is drastically inflated and ΔE values significantly exceeding −1000 mV are attained.

The two most extensively studied bi/confurcating flavoenzymes arguably are Nfn [27,36,53,117] and ETF [13,39,48,101,102,116,118,119,120]. These two systems are structurally only very distantly related [22] and in particular the binding pockets of their respective bi/confurcating flavins bear little to no similarities. It therefore is striking that in both enzymes an arginine residue is positioned with one of the NH-groups of the guanidino side chain in hydrogen bonding distance to N5 (Figure 13). These arginines are situated in completely unrelated (both with respect to sequence and to structure) polypeptide loops (Figure 13) and their presence in both enzyme families must therefore be assumed to result from convergent (i.e., function- rather than heredity-driven) evolution. Considering the pK values of the guanidino side chain as measured in peptides (pK around 12 [112]) and of the N5-proton in flavin_aq_ (as indicated in Figure 14, left side), an intriguing pattern of redox-dependent hydrogen bondings emerges. In both the oxidized and the semireduced states of the flavin, the pK values of the N5-proton are likely to be lower than that of the guanidino side chain. Under these conditions, the respective guanidino-nitrogen will be protonated which will sterically (and electrochemically) destabilize the proton on N5 (that is, further lower its pK values in the o- and the sr-states). By contrast, upon double reduction of the flavin, the reverse situation will prevail with N5 protonated and a downshift of the pK value of the guanidino-NH. This pattern of pK values therefore will lead to a rocking-proton phenomenon increasing the interval between pK_r_ on one and pK_sr_/pK_o_ on the other side, reminiscent of the situation in FNR where, however, the increase in ΔpK concerns pK_o_ with respect to pK_sr_/pK_r_.

Intrigued by this astonishing analogy between the bifurcating flavins in ETF and Nfn, we explored the effect of increasing the interval between pK_o_/pK_sr_ and pK_r_ of the N5-proton on the redox properties of the flavin. Figure 14 (right-hand side) shows the result of increasing this ΔpK(N5) by 10 units in simulations of Formula (3). Not only does this modification substantially inflate the volume of positive redox cooperativity in [pK(N1),pK(N3),pK(N5)]-space (cf. Figure 12), it also drastically lowers the absolute attainable values of ΔE = E_1_ − E_2_, now largely exceeding −1300 mV both in the above-mentioned (Section 3.6) core region of the inverted-potential manifold and in the centre of the arms (see top view in Figure 12d). Such pK values for N1 and N3 are in line with the structural idiosyncrasies of the two considered bifurcating enzymes.

The at first sight peculiar feature of an arginine residue in close proximity to N5 common to both Nfn and bifurcating ETF therefore finds its straightforward rationalization in the ability of this structural pattern to induce extremely strong positive redox cooperativity, indispensable to the proper functioning of bi/confurcating flavoenzymes.

Interestingly, in the 3D structure (pdb-entry 5ODC) of the heterodisulfide (Hdr) enzyme from *Methanothermococcus thermolithotrophicus* [121], a lysine residue (Lys409) occupies the space close to the flavin’s N5 nitrogen (with a distance in the range of 3.2 Å from N5 to the nitrogen atom of the lysine’s side chain). Considering that the pK value of lysine is in the region of 10 [112], this residue might also engage in rocking-proton phenomena very similar to what is described above and in Figure 13. The Hdr of *Methanothermococcus* indeed is an enzyme considered to bifurcate pairs of electrons at its flavin cofactor towards the reduction of the CoB-CoM heterodisulfide (exergonically) and towards CO_2_ reduction (endergonically). However, neither the degree of coupling of the two 1-electron transfers nor the redox behaviour of this enzyme’s flavin moiety are sufficiently well characterized to firmly integrate the enzyme into the analytical framework of this article. Nevertheless, the fact that unrelated folds and diverse residues (with comparable pK values) may induce rocking-proton behaviour leading to the extreme redox cooperativity required for strongly coupled electron bi/confurcation is tantalizing and merits empirical testing, e.g., via replacement of the respective residues.

### 3.8. Minor Conformational Changes During Enzyme Turnover May Substantially Alter Redox Behaviour

In the majority of flavoproteins studied so far, redox behaviour (that is, the sign and degree of redox cooperativity) appears to be a distinctive and rigid property of each enzyme. A corollary of the impact of pK variations of the flavin’s protonatable nitrogens on its redox properties consists in the possibility that in a given flavoprotein, redox behaviour may be altered, and even shifted back and forth between negative and positive cooperativity, by relatively minor conformational changes. These changes may be caused by structural modifications, potentially induced by the presence/absence of a substrate or by its redox state, altering pK values of one or of several of the three protonatable nitrogens in appropriate ways.

In a subgroup of electron bi/confurcating flavoenzymes, i.e., the NAD-dependent dehydrogenases [122], the existence of such flexible redox properties has indeed been proposed to play a role in their catalytic reaction schemes. The observation that in this subgroup, one of the two redox compounds donating/accepting electrons to/from the electron bi/confurcating enzyme is an obligatory 2-electron redox substrate (NAD^+^/NADH or NADP^+^/NADPH) is at odds with traditional notions of the mechanism of electron bi/confurcation since a single flavin cannot handle three electrons at a time. It therefore was proposed that the cofactor’s redox cooperativity may be altered during enzyme turnover [122] although there still is no convincing model for how the enzyme may function even assuming malleable redox cooperativity. Intriguingly, however, heterogenous redox behaviour was indeed observed for one member of the family of NADH-dependent dehydrogenases, that is, the NADH-dependent [FeFe]-hydrogenases [54]. Whereas in a subcomplex of the enzyme, the flavin was found to display strongly negative cooperativity and hence a highly stabilized sr-state, the entire enzyme does not appear to promote stabilization of such an sr-state. By contrast, addition of NAD or NADH induced the appearance of the EPR signatures of the semireduced flavin even in the entire enzyme (Zuchan, unpublished results). We therefore hypothesize that relatively localized and minor conformational changes may be able to substantially alter redox properties and that the resulting swap of redox cooperativity may be a crucial element of the reaction cycle of certain flavoenzymes (e.g., see [52]).

### 3.9. Adjusting the Absolute Value of the Average 2-Electron Redox Potential (E = (E_1_ − E_2_)/2) to the Specific Redox Regime of Each Case of Enzyme

Obviously, and as mentioned in several places above, differential binding affinities (the molecular roots of which potentially but not necessarily overlap with the described pK modifications, see below) also potentially modify apparent redox midpoint potentials of the cofactors [57,95]. Lower binding affinities for the more reduced forms will push the redox midpoint potentials to more negative values (with a decrease of 2*RT/F, i.e., roughly 59 mV at room temperatures, per order of magnitude of decrease in affinity) and vice versa. While redox-dependent binding affinities are frequently encountered with flavoenzymes (often manifesting as loss of the flavin cofactor during isolation procedures in certain redox states and hence imposing addition of excess exogenous flavin during purification steps [49,96,97,98,99,100]), the molecular bases of differential binding are not well understood. In addition to redox-dependent free energies of binding entailed by the presence/absence of H-bonds (a corollary of the phenomena discussed in this work), overall electrostatic attraction/repulsion exerted by the binding pocket [123], π-stacking interactions between aromatic side chains and the isoalloxazine moiety [80] or differential steric matches between the binding pocket and redox-dependent conformers of the flavin (e.g., resulting from bending or puckering [124,125]) may participate in this effect. In principle, electrostatic or steric effects might therefore also be means to influence redox properties of flavins in binding pockets.

However, in the large sample of flavoenzymes analyzed and described in this work, modifications of pK values (as supported by 3D-structural information) alone suffice to rationalize the empirically observed redox cooperativity of their flavin cofactors. This strongly indicates that tuning pK values is molecular evolution’s preferred tool for coaxing these cofactors into the specific regimes of redox cooperativity required for optimal functioning of each enzyme. Differential binding affinities, by contrast, appear to serve for shifting the resulting E_1,2_-vs-pH curves into the desired range of absolute redox potentials.

## 4. Discussion

As illustrated in this work, flavoenzymes display a bewildering variability of their redox properties, not only with respect to the absolute value of the range of electrochemical potentials they operate in but also concerning the relative position of their 1-electron transitions, i.e., their redox cooperativity. While the absolute value of their redox range needs to adapt to the reducing/oxidizing power of their respective redox substrates, the degree of cooperativity is tuned to optimize their specific mode of redox catalysis, that is, from single-electron transfer through gating and 2-electron reactions to electron bifurcation.

The impact of pK values of protonatable sites of the flavin cofactor on the redox midpoint potentials of the individual 1-electron transitions (as described by Formula (3)) is not a hypothesis but an ineluctable electrochemical fact. The cases of flavoenzymes dealt with in this work strongly suggest that it is this very influence of pKs on E_1,2_ which is exploited by natural selection to tune redox properties of a given flavoenzyme into the desirable regime of redox cooperativity. Formula (3) in principle allows for all three protonatable sites to shift the flavin cofactor around in pK space, back and forth between negative and positive cooperativity. Empirically (i.e., as suggested by real-world flavoenzymes), however, evolution mainly appears to tinker with the pK values of the N5-proton to achieve its electrochemical goals (with further minor adjustments based on the pKs of the N1 proton). The hydrogen-bonding interactions with the N3 proton were found to mainly serve for anchoring the isoalloxazine in its binding site [85] and to induce shifts in E_1_ and E_2_ much smaller than those arising from pK modifications of the N5-proton [87].

As becomes apparent from several of the cases discussed in this work, specific modifications of pKs (and of the interval between the pKs pertaining to the three redox states) are produced in the real-world flavoenzymes by interactions with different amino acid residues and using strongly dissimilar secondary structural arrangements. It therefore appears futile to search for unique structural motifs in trying to rationalize specific regimes of redox cooperativity. It furthermore does not seem far-fetched to us to assume corresponding phenomena to play a role in other enzyme families relying on 2-electron compounds such as on quinones or on molybdopterins [38].

## Data Availability

Data is contained within the article or Supplementary Materials.

## Supplementary Materials

The following supporting information can be downloaded at: https://www.mdpi.com/article/10.3390/life16081277/s1, Figure S1: Appearance of the E_1_/E_2_-planes upon modification of specific parameters; Figure S2: Animated version of Figure 12b; SI_main_E12_vs_pH_pK.cc: C++ source file; SI_main_all_pKs_4_voxel_plot.cc: C++ source file.

## References

[1] Blyth A.W. (1879). The composition of cow’s milk in health and disease. J. Chem. Soc. Trans..

[2] György P., Kuhn R., Wagner-Jauregg T. (1933). Das Vitamin B_2_. Naturwissenschaften.

[3] Kuhn R., Reinemund K. (1934). Über die Synthese des 6.7.9-Trimethyl-flavins (Lumi-lactoflavins). Berichte Der Dtsch. Chem. Ges. (A B Ser.).

[4] Karrer P., Schöpp K., Benz F., Pfaehler K. (1935). Synthesen von Flavinen III. Helv. Chim. Acta.

[5] Massey V. (2000). The chemical and biological versatility of riboflavin. Biochem. Soc. Trans..

[6] Neims A.H., Hellerman L. (1970). Flavoenzyme catalysis. Annu. Rev. Biochem..

[7] Singer T.P., Edmondson D.E. (1978). Flavoproteins. Methods Enzymol..

[8] Ghisla S., Massey V. (1989). Mechanisms of flavoprotein-catalyzed reactions. Eur. J. Biochem..

[9] Mathews F.S. (1991). New flavoenzymes. Curr. Opin. Struct. Biol..

[10] Fraaije M.W., Mattevi A. (2000). Flavoenzymes: Diverse catalysts with recurrent features. Trends Biochem. Sci..

[11] Williams R.E., Bruce N.C. (2002). ‘New uses for an old enzyme’—The old yellow enzyme family of flavoenzymes. Microbiology.

[12] Macheroux P., Kappes B., Ealick S.E. (2011). Flavogenomics—A genomic and structural view of flavin-dependent proteins. FEBS J..

[13] Sato K., Nishina Y., Shiga K. (2013). Interaction between NADH and electron-transferring flavoprotein from *Megasphaera elsdenii*. J. Biochem..

[14] Segal H.M., Spatzal T., Hill M.G., Udit A.K., Rees D.C. (2017). Electrochemical and structural characterization of *Azotobacter vinelandii* flavodoxin II. Protein Sci..

[15] Mayhew S.G. (1999). The effects of pH and semiquinone formation on the oxidation-reduction potentials of flavin mononucleotide. A reappraisal. Eur. J. Biochem..

[16] Herrmann G., Jayamani E., Mai G., Buckel W. (2008). Energy conservation via electron-transferring flavoprotein in anaerobic bacteria. J. Bacteriol..

[17] Li F., Hinderberger J., Seedorf H., Zhang J., Buckel W., Thauer R.K. (2008). Coupled ferredoxin and crotonyl coenzyme A (CoA) reduction with NADH catalyzed by the butyryl-CoA dehydrogenase/Etf complex from *Clostridium kluyveri*. J. Bacteriol..

[18] Buckel W., Thauer R.K. (2013). Energy conservation via electron bifurcating ferredoxin reduction and proton/Na+ translocating ferredoxin oxidation. Biochim. Biophys. Acta (BBA)-Bioenerg..

[19] Nitschke W., Russell M.J. (2012). Redox bifurcations: Mechanisms and importance to life now, and at its origin. BioEssays.

[20] Peters J.W., Miller A.F., Jones A.K., King P.W., Adams M.W. (2016). Electron bifurcation. Curr. Opin. Chem. Biol..

[21] Peters J.W., Beratan D.N., Bothner B., Dyer R.B., Harwood C.S., Heiden Z.M., Hille R., Jones A.K., King P.W., Lu Y. (2018). A new era for electron bifurcation. Curr. Opin. Chem. Biol..

[22] Baymann F., Schoepp-Cothenet B., Duval S., Guiral M., Brugna M., Baffert C., Russell M.J., Nitschke W. (2018). On the natural history of flavin-based electron bifurcation. Front. Microbiol..

[23] Brandt U. (1996). Energy conservation by bifurcated electron-transfer in the cytochrome-*bc*1 complex. Biochim. Biophys. Acta (BBA)-Bioenerg..

[24] Crofts A.R., Hong S., Wilson C., Burton R., Victoria D., Harrison C., Schulten K. (2013). The mechanism of ubihydroquinone oxidation at the Q_o_-site of the cytochrome *bc*_1_ complex. Biochim. Biophys. Acta.

[25] Bergdoll L., Ten Brink F., Nitschke W., Picot D., Baymann F. (2016). From low-to high-potential bioenergetic chains: Thermodynamic constraints of Q-cycle function. Biochim. Biophys. Acta (BBA)-Bioenerg..

[26] Wikström M.K.F., Berden J.A. (1972). Oxidoreduction of cytochrome b in the presence of antimycin. Biochim. Biophys. Acta (BBA)-Bioenerg..

[27] Lubner C.E., Jennings D.P., Mulder D.W., Schut G.J., Zadvornyy O.A., Hoben J.P., Tokmina-Lukaszewska M., Berry L., Nguyen D.M., Lipscomb G.L. (2017). Mechanistic insights into energy conservation by flavin-based electron bifurcation. Nat. Chem. Biol..

[28] Grein F., Ramos A.R., Venceslau S.S., Pereira I.A. (2013). Unifying concepts in anaerobic respiration: Insights from dissimilatory sulfur metabolism. Biochim. Biophys. Acta (BBA)-Bioenerg..

[29] Nitschke W., Schoepp-Cothenet B., Duval S., Zuchan K., Farr O., Baymann F., Panico F., Minguzzi A., Branscomb E., Russell M.J. (2022). Aqueous electrochemistry; the toolbox for life’s emergence from redox disequilibria. Electrochem. Sci. Adv..

[30] Martin W.F. (2012). Hydrogen, metals, bifurcating electrons, and proton gradients: The early evolution of biological energy conservation. FEBS Lett..

[31] Branscomb E., Russell M.J. (2013). Turnstiles and bifurcators: The disequilibrium converting engines that put metabolism on the road. Biochim. Biophys. Acta (BBA)-Bioenerg..

[32] Nitschke W., Farr O., Gaudu N., Truong C., Guyot F., Russell M.J., Duval S. (2024). The winding road from origin to emergence (of life). Life.

[33] Truong C., Gaudu N., Farr O., Clouet A., Ferry D., Guyot F., Ona-Nguema G., Ruby C., Nitschke W., Grauby O. (2025). Fe^2+^ disproportionation within iron-rich alkaline vent analogues reveals proto-bioenergetic systems. Nat. Commun..

[34] Kayastha K., Katsyv A., Himmrich C., Welsch S., Schuller J.M., Ermler U., Mueller V. (2021). Structure-based electron-confurcation mechanism of the Ldh-EtfAB complex. eLife.

[35] Yuly J.L., Zhang P., Beratan D.N. (2021). Energy transduction by reversible electron bifurcation. Curr. Opin. Electrochem..

[36] Yuly J.L., Lubner C.E., Zhang P., Beratan D.N., Peters J.W. (2019). Electron bifurcation: Progress and grand challenges. Chem. Commun..

[37] Terai K., Hjelmstad A.S., Beratan D.N. (2026). Inverted Potentials Enhance Electron Bifurcation Efficiency Prior to Steady State. J. Phys. Chem. Lett..

[38] Duval S., Santini J.M., Lemaire D., Chaspoul F., Russell M.J., Grimaldi S., Nitschke W., Schoepp-Cothenet B. (2016). The H-bond network surrounding the pyranopterins modulates redox cooperativity in the molybdenum-*bis*PGD cofactor in arsenite oxidase. Biochim. Biophys. Acta Bioenerg..

[39] Chowdhury N.P., Mowafy A.M., Demmer J.K., Upadhyay V., Koelzer S., Jayamani E., Kahnt J., Hornung M., Demmer U., Ermler U. (2014). Studies on the mechanism of electron bifurcation catalyzed by electron transferring flavoprotein (Etf) and butyryl-CoA dehydrogenase (Bcd) of *Acidaminococcus fermentans*. J. Biol. Chem..

[40] Chowdhury N.P., Klomann K., Seubert A., Buckel W. (2016). Reduction of flavodoxin by electron bifurcation and sodium ion-dependent reoxidation by NAD^+^ catalyzed by ferredoxin-NAD^+^ reductase (Rnf). J. Biol. Chem..

[41] Hauska G., Hurt E., Gabellini N., Lockau W. (1983). Comparative aspects of quinol-cytochrome c/plastocyanin oxidoreductases. Biochim. Biophys. Acta Rev. Bioenerg..

[42] Michaelis L., Schwarzenbach G. (1938). The intermediate forms of oxidation-reduction of the flavins. J.Biol.Chem..

[43] Ehrenberg A., Muller F., Hemmerich P. (1967). Basicity, visible spectra and electron spin resonance of flavosemiquinone anions. Eur. J. Biochem..

[44] Anderson R.F. (1983). Energetics of the one-electron reduction steps of riboflavin, FMN and FAD to their fully reduced forms. Biochim. Biophys. Acta.

[45] Furlan C., Chongdar N., Gupta P., Lubitz W., Ogata H., Blaza J.N., Birrell J.A. (2022). Structural insight on the mechanism of an electron-bifurcating [FeFe] hydrogenase. eLife.

[46] Kayastha K., Vitt S., Buckel W., Ermler U. (2021). Flavins in the electron bifurcation process. Arch. Biochem. Biophys..

[47] Stankovich M.T., Fox B.G. (1984). Redox potential-pH properties of the flavoprotein l-amino-acid oxidase. Biochim. Biophys. Acta (BBA)-Protein Struct. Mol. Enzymol..

[48] Pace C.P., Stankovich M.T. (1987). Redox properties of electron-transferring flavoprotein from *Megasphaera elsdenii*. Biochim. Biophys. Acta (BBA)-Protein Struct. Mol. Enzymol..

[49] Yoch D.C., Chen Y.P., Hardin M.G. (1990). Formate dehydrogenase from the methane oxidizer *Methylosinus trichosporium* OB3b. J. Bacteriol..

[50] Sled V.D., Rudnitzky N.I., Hatefi Y., Ohnishi T. (1994). Thermodynamic analysis of flavin in mitochondrial NADH: Ubiquinone oxidoreductase (complex I). Biochemistry.

[51] Sánchez-Azqueta A., Herguedas B., Hurtado-Guerrero R., Hervás M., Navarro J.A., Martínez-Júlvez M., Medina M. (2014). A hydrogen bond network in the active site of *Anabaena* ferredoxin-NADP+ reductase modulates its catalytic efficiency. Biochim. Biophys. Acta-Bioenerg..

[52] Hu J., Chuenchor W., Rokita S.E. (2015). A switch between one-and two-electron chemistry of the human flavoprotein iodotyrosine deiodinase is controlled by substrate. J. Biol. Chem..

[53] Wise C.E., Ledinina A.E., Mulder D.W., Chou K.J., Peters J.W., King P.W., Lubner C.E. (2022). An uncharacteristically low-potential flavin governs the energy landscape of electron bifurcation. Proc. Natl. Acad. Sci. USA.

[54] Zuchan K., Breuer N., Laurich C., Nitschke W., Baymann F., Birrell J.A. (2025). Thermodynamic landscape of the redox-centres in the electron-confurcating [FeFe]-Hydrogenase (TmHydABC) of *Thermotoga maritima*. Biochim. Biophys. Acta (BBA)-Bioenerg..

[55] Schopfer L.M., Ludwig M.L., Massey V., Curti B., Ronchi S., Zanetti G. (1991). A working proposal for the role of the apoprotein in determining the redox potentials of the flavin in flavoproteins: Correlations between potentials and flavin pKs. Flavins and Flavoproteins.

[56] Wardman P. (1989). Reduction potentials of one-electron couples involving free radicals in aqueous solution. J. Phys. Chem. Ref. Data.

[57] Clark W.M. (1960). Oxidation-Reduction Potentials of Organic Systems.

[58] Zu Y., Couture M.M.-J., Kolling D.R.J., Crofts A.R., Eltis L.D., Fee J.A., Hirst J. (2003). Reduction potentials of Rieske clusters: Importance of the coupling between oxidation state and histidine protonation state. Biochemistry.

[59] Agarwal R.G., Coste S.C., Groff B.D., Heuer A.M., Noh H., Parada G.A., Wise C.F., Nichols E.M., Warren J.J., Mayer J.M. (2022). Free Energies of Proton-Coupled Electron Transfer Reagents and Their Applications. Chem. Rev..

[60] Hsueh K.-L., Wrestler W.M., Markley J.L. (2010). NMR investigations of the Rieske protein from Thermus thermophilus support a coupled proton and electron transfer mechanism. J. Am. Chem. Soc..

[61] Jolivet J.-P., Tronc E., Chanéac C. (2006). Iron oxides: From molecular clusters to solid. A nice example of chemical versatility. C. R. Geosci..

[62] Moonen C.T.W., Verwoort J., Müller F. (1984). Reinvestigation of the structure of oxidized and reduced flavin: Carbon-13 and nitrogen-15 nuclear magnetic resonance study. Biochemistry.

[63] Moonen C.T.W., Verwoort J., Müller F. (1984). Carbon-13 nuclear magnetic resonance study on the dynamics of the conformation of reduced flavin. Biochemistry.

[64] Müller F., Weber S., Schleicher E. (2014). NMR spectroscopy on flavins and flavoproteins. Flavins and Flavoproteins: Methods and Protocols.

[65] Michaelis L., Schubert M.P., Smythe C.V. (1936). Potentiometric study of the flavins. J. Biol. Chem..

[66] Draper R.D., Ingraham L.L. (1968). A potentiometric study of the flavin semiquinone equilibrium. Arch. Biochem. Biophys..

[67] Beinert H. (1956). Spectral characteristics of flavins at the semiquinoid oxidation level. J. Am. Chem. Soc..

[68] Massey V., Palmer G. (1966). On the existence of spectrally distinct classes of flavoprotein semiquinones. A new method for the quantitative production of flavoprotein semiquinones. Biochemistry.

[69] Zavoisky E. (1945). Spin-magnetic resonance in paramagnetics. J. Phys. (USSR).

[70] Bleaney B. (1951). Hyperfine structure in paramagnetic salts and nuclear alignment. Lond. Edinb. Dublin Philos. Mag. J. Sci..

[71] Edmondson D.E. (1985). Electron-spin-resonance studies on flavoenzymes. Biochem. Soc. Trans..

[72] Robertson D.E., Prince R.C., Bowyer J.R., Matsuura K., Dutton P.L., Ohnishi T. (1984). Thermodynamic properties of the semiquinone and its binding site in the ubiquinol-cytochrome c (c2) oxidoreductase of respiratory and photosynthetic systems. J. Biol. Chem..

[73] Wu S.Y., Rothery R.A., Weiner J.H. (2015). Pyranopterin coordination controls molybdenum electrochemistry in *Escherichia coli* nitrate reductase. J. Biol. Chem..

[74] Niemz A., Imbriglio J., Rotello V.M. (1997). Model systems for flavoenzyme activity: One-and two-electron reduction of flavins in aprotic hydrophobic environments. J. Am. Chem. Soc..

[75] Cerda J.F., Koder R.L., Lichtenstein B.R., Moser C.M., Miller A.-F., Dutton P.L. (2008). Hydrogen bond-free flavin redox properties: Managing flavins in extreme aprotic solvents. Org. Biomol. Chem..

[76] Tan S.L., Kan J.M., Webster R.D. (2013). Differences in proton-coupled electron-transfer reactions of flavin mononucleotide (FMN) and flavin adenine dinucleotide (FAD) between buffered and unbuffered aqueous solutions. J. Phys. Chem. B.

[77] Tan S.L., Novianti M.L., Webster R.D. (2015). Effects of low to intermediate water concentrations on proton-coupled electron transfer (PCET) reactions of flavins in aprotic solvents and a comparison with the PCET reactions of quinones. J. Phys. Chem. B.

[78] Mayhew S.G. (1971). Studies on flavin binding in flavodoxins. Biochim. Biophys. Acta (BBA)-Enzymol..

[79] Mayhew S.G., Ludwig M.L., Boyer P.D. (1975). Flavodoxins and electron-transferring flavoproteins. The Enzymes.

[80] Zhou Z., Swenson R.P. (1996). The cumulative electrostatic effect of aromatic stacking interactions and the negative electrostatic environment of the flavin mononucleotide binding site is a major determinant of the reduction potential for the flavodoxin from *Desulfovibrio vulgaris* [Hildenborough]. Biochemistry.

[81] Steensma E., Heering H.A., Hagen W.R., van Mierlo C.P. (1996). Redox properties of wild-type, Cys69Ala, and Cys69Ser *Azotobacter vinelandii* flavodoxin II as measured by cyclic voltammetry and EPR spectroscopy. Eur. J. Biochem..

[82] Ludwig M.L., Pattridge K.A., Metzger A.L., Dixon M.M., Eren M., Feng Y., Swenson R.P. (1997). Control of oxidation-reduction potentials in flavodoxin from *Clostridium beijerinckii*: The role of conformation changes. Biochemistry.

[83] O’Farrell P.A., Walsh M.A., McCarthy A.A., Higgins T.M., Voordouw G., Mayhew S.G. (1998). Modulation of the Redox Potentials of FMN in *Desulfovibrio vulgaris* Flavodoxin: Thermodynamic Properties and Crystal Structures of Glycine-61 Mutants. Biochemistry.

[84] Yalloway G.N., Mayhew S.G., Malthouse J.P.G., Gallagher M.E., Curley G.P. (1999). pH-dependent spectroscopic changes associated with the hydroquinone of FMN in flavodoxins. Biochemistry.

[85] Bradley L.H., Swenson R.P. (1999). Role of glutamate-59 hydrogen bonded to N(3)H of the flavin mononucleotide cofactor in the modulation of the redox potentials of the *Clostridium beijerinckii* flavodoxin. Glutamate-59 is not responsible for the pH dependency but contributes to the stabilization of the flavin semiquinone. Biochemistry.

[86] Chang F.C., Swenson R.P. (1999). The Midpoint Potentials for the oxidized-semiquinone couple for Gly57 mutants of the *Clostridium Beijerinckii* flavodoxin correlate with changes in the hydrogen-bonding interaction with the proton on N(5) of the reduced flavin mononucleotide cofactor as measured by NMR chemical shift temperature dependencies. Biochemistry.

[87] Bradley L.H., Swenson R.P. (2001). Role of hydrogen bonding interactions to N(3)H of the flavin mononucleotide cofactor in the modulation of the redox potentials of the *Clostridium beijerinckii* flavodoxin. Biochemistry.

[88] McCarthy A.A., Walsh M.A., Verma C.S., O’Connell D.P., Reinhold M., Yalloway G.N., d’Arcy D., Higgins T.M., Voordouw G., Mayhew S.G. (2002). Crystallographic investigation of the role of aspartate 95 in the modulation of the redox potentials of *Desulfovibrio vulgaris* flavodoxin. Biochemistry.

[89] Ishikita H. (2008). Redox potential difference between *Desulfovibrio vulgaris* and *Clostridium beijerinckii* flavodoxins. Biochemistry.

[90] Mayhew S.G., Foust G.P., Massey V. (1969). Oxidation-reduction properties of flavodoxin from *Peptostreptococcus elsdenii*. J. Biol. Chem..

[91] Müller F., Hemmerich P., Ehrenberg A., Palmer G., Massey V. (1970). The chemical and electronic structure of the neutral flavin radical as revealed by electron spin resonance spectroscopy of chemically and isotopically substituted derivatives. Eur. J. Biochem..

[92] Medina M., Gomez-Moreno C., Cammack R. (1995). Electron spin resonance and electron nuclear double resonance studies of flavoproteins involved in the photosynthetic electron transport in the cyanobacterium Anabaena sp. PCC 7119. Eur. J. Biochem..

[93] Watt W., Tulinsky A., Swenson R.P., Watenpaugh K.D. (1991). Comparison of the crystal structures of a flavodoxin in its three oxidation states at cryogenic temperatures. J. Mol. Biol..

[94] Sancho J. (2006). Flavodoxins: Sequence, folding, binding, function and beyond. Cell. Mol. Life Sci..

[95] Dutton P.L. (1978). Redox potentiometry: Determination of midpoint potentials of oxidation-reduction components of biological electron-transfer systems. Methods Enzymol..

[96] Verhagen M.F., O’Rourke T., Adams M.W. (1999). The hyperthermophilic bacterium, *Thermotoga maritima*, contains an unusually complex iron-hydrogenase: Amino acid sequence analyses versus biochemical characterization. Biochim. Biophys. Acta (BBA)-Bioenerg..

[97] Schuchmann K., Müller V. (2012). A bacterial electron-bifurcating hydrogenase. J. Biol. Chem..

[98] Wang S., Huang H., Kahnt J., Mueller A.P., Köpke M., Thauer R.K. (2013). NADP-specific electron-bifurcating [FeFe]-hydrogenase in a functional complex with formate dehydrogenase in *Clostridium autoethanogenum* grown on CO. J. Bacteriol..

[99] Kpebe A., Benvenuti M., Guendon C., Rebai A., Fernandez V., Le Laz S., Etienne E., Guigliarelli B., García-Molina G., de Lacey A.L. (2018). A new mechanistic model for an O_2_-protected electron-bifurcating hydrogenase, Hnd from *Desulfovibrio fructosovorans*. Biochim. Biophys. Acta (BBA)-Bioenerg..

[100] Chongdar N., Pawlak K., Rüdiger O., Reijerse E.J., Rodríguez-Maciá P., Lubitz W., Birrell J.A., Ogata H. (2020). Spectroscopic and biochemical insight into an electron-bifurcating [FeFe] hydrogenase. JBIC J. Biol. Inorg. Chem..

[101] Garcia Costas A.M., Poudel S., Miller A.F., Schut G.J., Ledbetter R.N., Fixen K.R., Seefeldt L.C., Adams M.W., Harwood C.S., Boyd E.S. (2017). Defining electron bifurcation in the electron-transferring flavoprotein family. J. Bacteriol..

[102] Byron C.M., Stankovich M.T., Husain M., Davidson V.L. (1989). Unusual redox properties of electron-transfer flavoprotein from *Methylophilus methylotrophus*. Biochemistry.

[103] Yang K.Y., Swenson R.P. (2007). Modulation of the redox properties of the flavin cofactor through hydrogen-bonding interactions with the N (5) atom: Role of αSer254 in the electron-transfer flavoprotein from the methylotrophic bacterium W3A1. Biochemistry.

[104] Tsai M.H., Saier M.H. (1995). Phylogenetic characterization of the ubiquitous electron transfer flavoprotein families ETF-α and ETF-β. Res. Microbiol..

[105] Das D., Miller A.-F. (2024). A single hydrogen bond that tunes flavin redox reactivity and activates it for modification. Chem. Sci..

[106] De Causmaecker S., Douglass J.S., Fantuzzi A., Nitschke W., Rutherford A.W. (2019). Energetics of the exchangeable quinone, QB, in Photosystem II. Proc. Natl. Acad. Sci. USA.

[107] Corrado M.E., Aliverti A., Zanetti G., Mayhew S.G. (1996). Analysis of the oxidation-reduction potentials of recombinant ferredoxin-NADP+ reductase from spinach chloroplasts. Eur. J. Biochem..

[108] Bruns C.M., Karplus A.P. (1995). Refined crystal structure of spinach ferredoxin reductase at 1.7 Å resolution: Oxidized, reduced and 2′-phospho-5′-AMP bound states. J. Mol. Biol..

[109] Faro M., Gómez-Moreno C., Stankovich M., Medina M. (2002). Role of critical charged residues in reduction potential modulation of ferredoxin-NADP+ reductase: Differential stabilization of FAD redox forms. Eur. J. Biochem..

[110] Carrillo N., Ceccarelli E.A. (2003). Open questions in ferredoxin-NADP+ reductase catalytic mechanism. Eur. J. Biochem..

[111] Dumit V.I., Essigke T., Cortez N., Ullmann G.M. (2010). Mechanistic insights into ferredoxin–NADP (H) reductase catalysis involving the conserved glutamate in the active site. J. Mol. Biol..

[112] Thurlkill R.L., Grimsley G.R., Scholtz J.M., Pace C.N. (2006). pK values of the ionizable groups of proteins. Protein Sci..

[113] Saito K., Sakashita N., Ishikita H. (2016). Energetics of the proton transfer pathway for tyrosine D in photosystem II. Aust. J. Chem..

[114] Saito K., Shen J.R., Ishida T., Ishikita H. (2011). Short hydrogen bond between redox-active tyrosine YZ and D1-His190 in the photosystem II crystal structure. Biochemistry.

[115] Zhou Z., Chen Z., Kang X.W., Zhou Y., Wang B., Tang S., Zou S., Zhang Y., Hu Q., Bai F. (2022). The nature of proton-coupled electron transfer in a blue light using flavin domain. Proc. Natl. Acad. Sci. USA.

[116] Demmer J.K., Pal Chowdhury N., Selmer T., Ermler U., Buckel W. (2017). The semiquinone swing in the bifurcating electron transferring flavoprotein/butyryl-CoA dehydrogenase complex from *Clostridium difficile*. Nat. Commun..

[117] Demmer J.K., Huang H., Wang S., Demmer U., Thauer R.K., Ermler U. (2015). Insights into flavin-based electron bifurcation via the NADH-dependent reduced ferredoxin:NADP oxidoreductase structure. J. Biol. Chem..

[118] Sucharitakul J., Buckel W., Chaiyen P. (2021). Rapid kinetics reveal surprising flavin chemistry in bifurcating electron transfer flavoprotein from *Acidaminococcus fermentans*. J. Biol. Chem..

[119] Duan H.D., Khan S.A., Miller A.F. (2021). Photogeneration and reactivity of flavin anionic semiquinone in a bifurcating electron transfer flavoprotein. Biochim. Biophys. Acta (BBA)-Bioenerg..

[120] González-Viegas M., Kar R.K., Miller A.F., Mroginski M.A. (2023). Noncovalent interactions that tune the reactivities of the flavins in bifurcating electron transferring flavoprotein. J. Biol. Chem..

[121] Wagner T., Koch J., Ermler U., Shima S. (2017). Methanogenic heterodisulfide reductase (HdrABC-MvhAGD) uses two noncubane [4Fe-4S] clusters for reduction. Science.

[122] Zuchan K., Baymann F., Baffert C., Brugna M., Nitschke W. (2021). The dyad of the Y-junction-and a flavin module unites diverse redox enzymes. Biochim. Biophys. Acta (BBA)-Bioenerg..

[123] Zhou Z., Swenson R.P. (1995). Electrostatic effects of surface acidic amino acid residues on the oxidation-reduction potentials of the flavodoxin from *Desulfovibrio vulgaris* (Hildenborough). Biochemistry.

[124] Zheng Y.J., Ornstein R.L. (1996). A theoretical study of the structures of flavin in different oxidation and protonation states. J. Am. Chem. Soc..

[125] Kar R.K., Miller A.F., Mroginski M.A. (2022). Understanding flavin electronic structure and spectra. Wiley Interdiscip. Rev. Comput. Mol. Sci..

