# Supplementary material for: pK Values of the Cofactor Tune the Redox Regime of Flavoenzymes
Source: Life (Basel). 2026 Jul 31;16(8):1277. doi: 10.3390/life16081277 (PMC13514486; doi:10.3390/life16081277)
Supplement: Supplementary file 1 [file life-16-01277-s001.zip › Fig_SI_1.pdf]

pK$_o$(N5) fixed -0.5, pK$_{sr}$/pK$_r$ floating
all other pKs as in flavin$_{aq}$

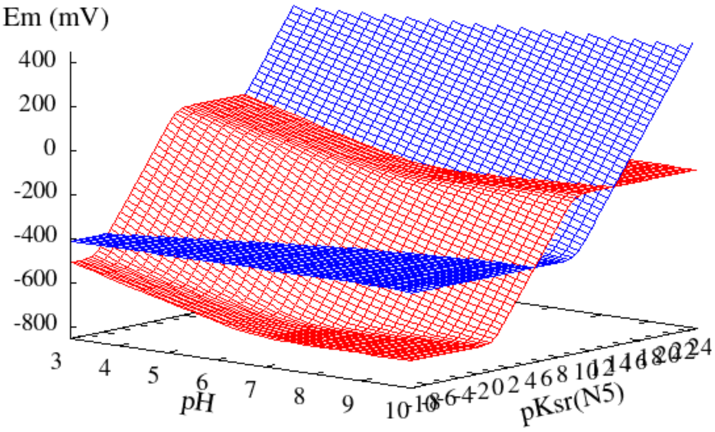

all pKs(N5) floating, ΔpKs(N5) as in flavin$_{aq}$
all other pKs as in flavin$_{aq}$

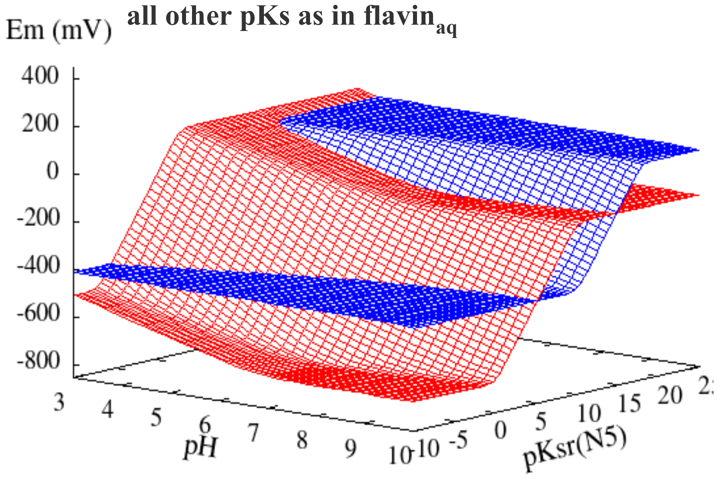

all pKs(N5) floating, ΔpKs(N5) as in flavin$_{aq}$
all pKs(N1) down by 4; all pKs(N3) as in flavin$_{aq}$

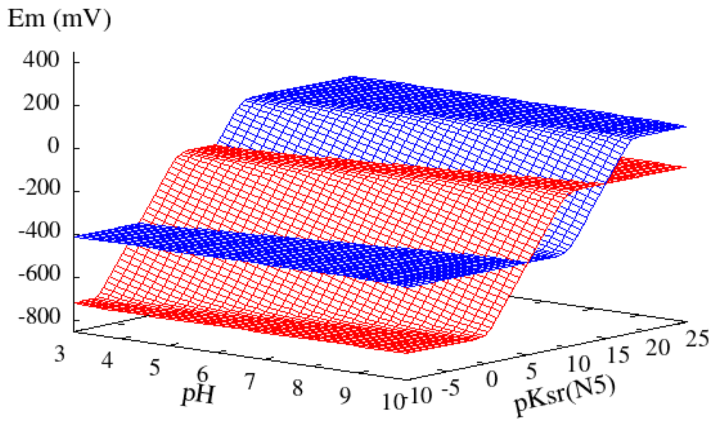

all pKs(N5) floating, ΔpKs(N5) as in flavin$_{aq}$
all pKs(N1) up by 4; all pKs(N3) as in flavin$_{aq}$

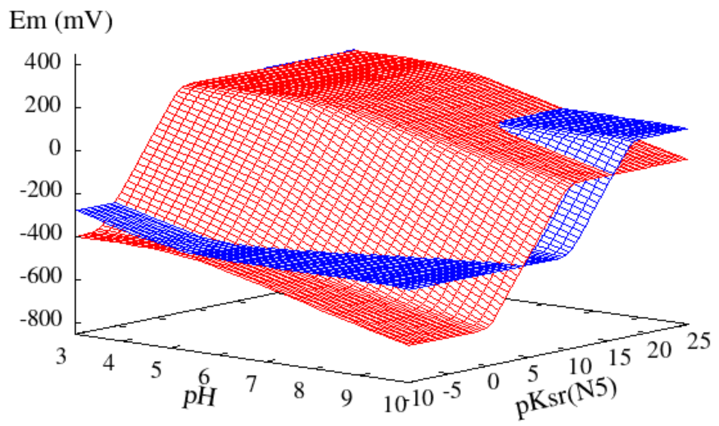

all pKs(N5) floating, ΔpKs(N5) as in flavin$_{aq}$
all pKs(N3) down by 4; all pKs(N1) as in flavin$_{aq}$

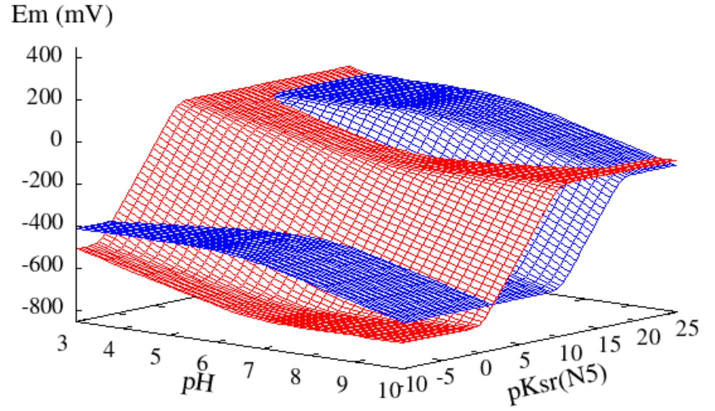

all pKs(N5) floating, ΔpKs(N5) = 6
all other pKs as in flavin$_{aq}$

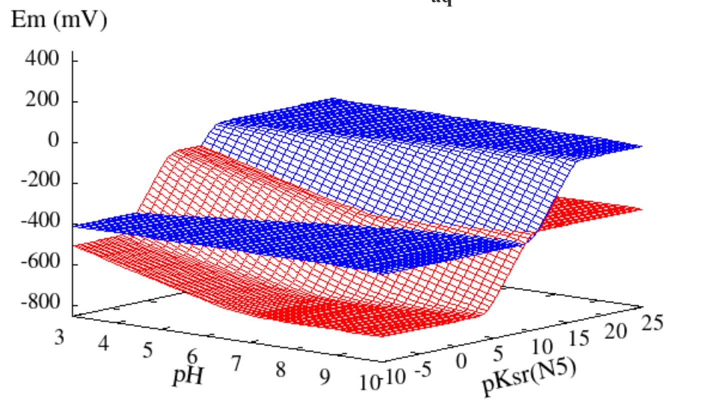

Legend to Figure S1: Appearance of the E$_1$/E$_2$-planes upon modification of specific parameters, e.g. absolute values of N1/N3/N5 or gaps between pK$_o$, pK$_{sr}$ and pK$_r$
